# Use of complementary and alternative medicine in children affected by oncologic, neurologic and liver diseases: a narrative review

**DOI:** 10.1186/s13052-023-01554-0

**Published:** 2023-11-15

**Authors:** Francesca Casini, Francesca Scaltrito, Maria Teresa Grimaldi, Tudor Lucian Pop, Valeria Calcaterra, Gian Vincenzo Zuccotti, Massimo Pettoello-Mantovani, Pietro Ferrara, Giovanni Corsello, Valentina Fabiano

**Affiliations:** 1 Pediatric Department, University of Milan, “V. Buzzi” Children’s Hospital, 20154 Milan, Italy; 2 Department of Medical and Surgical Sciences, University of Foggia, Foggia, Italy; 3 2Nd Pediatric Discipline, Department of Mother and Child, Center of Expertise in Pediatric Liver Rare Diseases, Iuliu Hatieganu University of Medicine and Pharmacy2Nd Pediatric ClinicEmergency Clinical Hospital for Children Cluj-Napoca, Cluj-Napoca, Romania; 4 European Pediatric Association-Union of National European Pediatric Societies and Associations, Berlin, Germany; 5 Department of Internal Medicine, University of Pavia, 27100 Pavia, Italy; 6 Department of Biomedical and Clinical Sciences, University of Milan, 20157 Milan, Italy; 7 Department of Medicine and Surgery, University Campus Bio-Medico, Rome, Italy; 8 Operative Research Unit of Pediatrics, Fondazione Policlinico Universitario Campus Bio-Medico, Rome, Italy; 9 Department of Pediatrics, University of Palermo, Palermo, Italy

**Keywords:** Alternative and complementary medicine, Children, Medications, Oncology, Neurology, Liver diseases

## Abstract

Complementary and alternative medicine (CAM) consist of a broad group of restorative resources often linked to existing local cultures and established health care systems and are also increasingly used in children with some serious illnesses. In this narrative review, we examine the epidemiology of the use, efficacy, and safety of complementary and alternative medicine in pediatric oncology, neurology, and hepatology. We searched for relevant articles published in Pubmed evaluating CAM use and its efficacy in safety in children affected by oncologic, neurologic and liver diseases**.** CAM is used to improve the success of conventional therapies, but also to alleviate the pain, discomfort, and suffering resulting from the diseases and their treatment, which are often associated with a significant burden of adverse effects. CAM use must be evaluated in children with neurological, oncological and liver diseases.

## Introduction

The term complementary therapy is used in conjunction with alternative therapy to describe medical procedures, based on the connection between body and mind, which can help patients affected by severe health conditions feel better and improve their quality of life. The use of complementary and alternative medicine in pediatric oncology has increased significantly in recent years. In the 1970s, when these disciplines first appeared on the health care scene, they were provided primarily as an alternative to conventional health care and thus came to be known collectively as "alternative medicine." The name "complementary medicine" developed when the two systems began to be used alongside each other (to "complement"). Over the years, the term "complementary" has moved from describing this relationship between unconventional health disciplines and conventional care to defining the group of disciplines themselves. This change and overlapping terminology may explain some of the confusion surrounding the topic [1].

Currently, the terms are often used in one combined definition, complementary and alternative medicine (CAM), although substantial differences exist between the two. Complementary medicine is increasingly being adopted as supplementary therapeutic modalities supporting standard therapies to relieve symptoms, while alternative medicine is used instead of conventional treatments [1]. However, replacing standard care with alternative treatments can expose patients to life-threatening consequences. In contrast, complementary therapies taken together with standard medical care can help patients better manage symptoms caused by cancer or mitigate side effects associated with therapy [2].

CAM includes a variety of health care practices summarized in Table 1. According to Cochrane, CAM consists of a broad group of restorative resources, which may involve therapeutic methods and practices and related theories and beliefs, often linked to existing local cultures and established health care systems [3].

**Table 1:** The most commonly used complementary and alternative medicines in patients with severe health conditions

Major complementary/alternative therapies (CAM) frequently used in patients with severe health conditions	
• Acupressure • Acupuncture • Applied kinesiology • Aromatherapy • Ayurveda • Biofeedback • Chiropractic • Dietary changes • Herbal medicine • Homoeopathy • Hypnosis	• Lifestyle changes • Massage • Meditation techniques • Nutritional therapy • Osteopathy • Physical activity • Reflexology • Self-help groups • Stress management techniques • Shiatsu • Yoga

In summary, the purpose of using complementary therapies in clinical practice is to improve the quality of the therapeutic environment and therapeutic relationships, with the goal of optimizing the patient's ability to heal, in conjunction with traditional therapies [4]. However, there are still many gaps in scientific research on complementary therapies, and the impact caused by the side effects of various CAM therapies may be underestimated due to the lack of comprehensive data. This paucity of data may be due to a lack of funding or commercial interest of pharmaceutical companies and the scarcity of adequately trained professionals on the use of CAM and the performance and interpretation of systematic reviews and methodological issues [4–7].

This informational article briefly discusses the use of CAM in pediatric patients affected by some serious, chronic and disabling diseases, specifically focusing on oncological, neurological, and liver diseases. In fact, many complementary and alternative medicines claim to relieve symptoms in cancer patients or during cancer treatments, reduce the burden of neurological conditions, relieve symptoms of liver disease, or reduce the risk of developing liver diseases. This article aims to further raise awareness among general pediatricians about the importance and risks of complementary therapies in clinical practice.

## Materials and methods

This is a brief narrative review that focuses on the use of CAM in pediatric patients with oncological, neurological, and liver conditions. Article selection was performed using established methods as described elsewhere [8]. In this article the authors summarize key information on the use of CAM in pediatrics obtained from scientific articles published during the past 20 years, including original studies, systematic review and meta-analysis. Articles search was performed using top academic search engines [8], including the classic academic databases Web of Science, Science.gov, Core, Scopus and PubMed, the search engine of the United States National Library of Medicine. Inclusion criteria involved all peer-reviewed articles published in English language, limited to child studies and published since 2003 (20 years). There was no geographical limitation for the articles considered for the review.

### Overview on complementary and alternative medicine

#### Therapeutic use of CAM

Many CAM practices have already been used for years, and their beneficial effects on the mind and body have been known since ancient times. In recent years, evidence has increased that these practices can play an important role in the treatment of some specific diseases, including cancer, neurological and liver diseases [1, 9].

One element shared by most complementary therapies is the multifactorial, multilevel view of human disease. According to complementary disciplines, the disease is no longer seen as a single pathological process but as a collection of physical, mental, social, and spiritual disorders. They also emphasize the human body's ability to recover faster in comfortable situations and under appropriate conditions.

Based on this holistic approach, complementary therapies aim to heal individuals by restoring their physical and inner balance. The goal is to stimulate and facilitate the body's positive responses in association with, not as an alternative to, "conventional" therapies, rather than targeting individual disease processes and troublesome symptoms [6].

### Efficacy of CAM

Numerous controlled clinical trials have demonstrated the usefulness of complementary therapies in treating various diseases with significant public health impact. The importance of CAM is further emphasized by the U.S. National Institutes of Health (NIH), which established the Office of Alternative Medicine, later named the National Center for Complementary and Integrative Health (NCCIH), to study the efficacy and safety of alternative therapies [7].

A wealth of information on the effectiveness of complementary and alternative medicine is available in the literature, including peer-reviewed publications, evidence-based reviews, expert group papers, and authoritative textbooks. However, no consensus has been reached on its effectiveness. Many CAM procedures have been studied and found to be effective in combination with conventional treatments, while other studies have found CAM to be ineffective or have reported contradictory and inconsistent results [9].

Standardization of data in CAM studies is difficult, which may explain the difficulty in reaching a consensus on their use in combination with the treatment of many conditions. In fact, complementary therapies cannot be standardized for individual conditions because, in most cases, their use is based on the patient's characteristics or experiences rather than on a clinically diagnosed disease in the traditional way. Outcomes are also difficult to standardize because they are often specific to individuals rather than based on objective, uniform measures such as blood pressure, blood glucose and inflammation indices. In addition, many studies lack a placebo control, which precludes any reliable conclusions. However, despite the lack of consensus on the efficacy of CAM, many studies have provided substantial data in favor of their use as integrated treatments in various serious disease conditions [9, 10].

### Safety of CAM

Unlike conventional medical treatments, which are thoroughly tested and regulated, most complementary therapies have not yet been sufficiently tested for their safety. Some studies have examined suspected CAM-related adverse reactions in the pediatric population and have warned of the risks and dangers of CAM in children, especially in "fragile" patients, such as those with cancer or neurology and liver diseases [11]. Studies have also provided insights into factors that might increase the risk of serious adverse reactions associated with the use of CAM in children. Indeed, their common description as natural remedies may suggest the assumption of safety, whereas the potential effects of CAM may instead represent an increased iatrogenic risk. In particular, the use of products containing more than two components and administered concurrently with conventional medications may pose a potential risk in younger patients [11], because responses to standard treatments in this population are often unpredictable and individual-based, and complementary treatments are usually not standardized [5, 7]. Adverse effects have also been reported in the use of herbal dietary supplements (HDS), as they can affect different physiological systems [12]. Studies on the adverse effects of CAM are significantly advancing knowledge in this area. However, health care providers should familiarize themselves with CAM practices and carefully balance the associated benefits and risks to best care for their patients.

### Use of complementary therapies in oncological diseases

Complementary therapies are used effectively in children with slow-moving forms of cancer to help cure or alleviate symptoms. Preferably, integrative pediatric oncology should be provided in pediatric hospitals or medical centers that participate in clinical trials or belong to pediatric oncology networks [13]. Complementary therapies, which in general are usually used in cancer patients, are summarized in Table 2.

**Table 2:** Complementary therapies frequently used in cancer patients

Complementary therapies frequently used in cancer patients
• Touch therapies: acupuncture, aromatherapy, reflexology, and massage
• Mind–body therapies: relaxation, guided imagery and hypnosis, yoga, meditation, tai chi
• Energy therapies: Reiki, therapeutic touch, and healing touch
• Talking therapies: trained counsellors (one-to-one) or group sharing experiences
• Changes in Daily lifestyle: physical activity, healthy diet, vitamins, probiotics, herbalism

The inclusion under the term CAM of several and various types of complementary practicesused for pediatric cancer patients may explain the wide range of prevalence, depending on the country (6%-91%), reported in a recent systematic review [13]. In particular, large differences have been reported between North America and Europe, which may also have been influenced by the time period over the past 30 years when CAM alone or CAM has been introduced in different countries, either in support of or as an alternative to cancer therapy [14, 15].

### The increased use of CAM to relieve symptoms in oncology patients

Parents of pediatric oncology patients are inclined to introduce unconventional treatments to reduce harming symptoms and alleviate complications of therapy[14]. The most common symptoms during cancer therapy are nausea and vomiting, whose multifactorial origin is typically related to chemotherapy [14, 16]. According to a 2022 review, CAM practices have helped to alleviate nausea, vomiting, mucositis, weight loss, anxiety, pain, and, most importantly, to improve children's quality of life [13].

The routine use of CAM, in addition to standard cancer therapies, has significantly increased during the past recent years by families and caregivers of cancer patients. Currently, these therapies are often an integral part of supportive care, especially used to control the side effects of cancer therapies. However, the use of CAM in cancer therapy is still debated, and there is no final consensus regarding its effectiveness and safety [17].

Different types of complementary therapies are used during cancer treatment. The National Center for Complementary and Alternative Medicine (NCCAM), popular categories of CAM are natural products, including plants/herbs, a practice also known as "herbalism," vitamins and other dietary supplements, mind–body practices (prayer, meditation, yoga, acupuncture, guided imagery, hypnotherapy, tai chi) manipulative practices (e.g., massage, chiropractic), new "biological field therapies" (e.g., Reiki, healing touch, qi gong), traditional healers, and other medical practices such as Ayurvedic medicine or traditional Chinese medicine, when used as a support to conventional medicine [18]. Homeopathy, dietary treatments and nutritional supplements seem very popular in Germany [7], while herbal extracts are mostly used in Mexico and water therapy and Spirulina in Malaysia[17]. Although often used during cancer treatments, chiropractic care needs further studies to demonstrate its effectiveness in pediatric oncology[19]. A recent review reports that several studies first observed the effects of CAM modalities on symptoms related to cancer therapy, and that hypnosis, imagery/visualization and music therapy were found to be most useful in relieving procedure-related pain [19].

This section briefly discusses some of the most commonly used CAM procedures in pediatric oncology.

### Massage

One of the most used CAM interventions for children with cancer is massage. An extensive review of massage practice in pediatric patients, which reports 24 randomized controlled trials between 1992 and 2006, found that massage was strongly effective on anxiety in children, especially after multiple sessions of this intervention [20]. In addition, evidence has been reported of other side symptoms relieved by massage, including nausea, pain, depression, stress, anger, and fatigue [21–23]. A study on a limited cohort of pediatric oncology patients of different ages and mixed diagnosesreported positive results in reducing heart rate and anxiety and very positive evaluations of the massage experience by the participants [24].

Studies on massage in children undergoing bone marrow transplantation (BMT) are scarce. In one study, fifty young cancer patients undergoing BMT received professional massage, parental massage, or constituted the control group. There was a significant difference in incision days after BMT in the combined group that received massage (parental and professional). A significant reduction in anxiety and immediate discomfort was seen in the group that received professional massage [21]. Another follow-up study conducted by the same team found no differences in depression, quality of life, or posttraumatic stress between a child intervention group (humor and massage), a parent intervention group (massage and relaxation), and a control group receiving only standard care, although an improvement and adjustment of symptoms were observed in all groups [25]. In a smaller pilot study, the combination of tri-weekly massage and acupressure versus standard care demonstrated benefits in terms of nausea, fatigue, pain, and reduction of mucositis and benefits were reported by caregivers using this practice [24].

### Yoga

There is evidence that yoga improves physical strength and flexibility as well as mental health through toning, stretching, and relaxation exercises. It has also been shown to beneficially influence the autonomic nervous system [26] by decreasing salivary cortisol levels, plasma renin levels, and urinary norepinephrine and epinephrine levels, as well as reducing blood pressure and heart rate [27]. In two studies yoga has been shown to be safe and feasible in pediatric cancer patients undergoing chemotherapy [28, 29]. However, further research is needed to demonstrate its efficacy in effectively controlling the symptom.

A recent study reports that a significant improvement in pain was achieved in patients using yoga regardless of their age. In addition, this study also reports the benefits of yoga in parents caring for their children. It was shown that one yoga experience was important enough for parents to control emotions and contain anxiety. In summary, this study emphasized that it was feasible for children and adolescents with hematologic or oncologic disease and their parents to participate in the yoga intervention [30]. Similarly, a different study suggested that patients' guardians experienced a significant decrease in anxiety after using relaxation practices [31]. Finally, yoga has been shown to be significantly effective in reducing fatigue symptoms in pediatric brain tumor patients and in improving their sleep.

### Acupuncture and acupressure

Acupuncture includes a group of techniques in which small needles, heat or electrical stimulation are placed at specific anatomical points. Acupuncture points are located on meridians along which qi (a "life energy") flows, while acupressure uses pressure applied with hands or other devices on the same acupuncture points [32]. Children have been shown to tolerate acupuncture well [33] and to have no bleeding problems. In addition, acupuncture has been reported to be effective in reducing chemotherapy-induced nausea and vomiting during or at the end of chemotherapy treatment [34]. In a meta-analysis that evaluated the effects of acupuncture on postoperative nausea and vomiting, acupuncture was shown to reduce vomiting and nausea [34].

Acupressure is similar to acupuncture but uses pressure instead of needles. This practice can be helpful for children who suffer from agophobia or when an acupuncture expert is not accessible. The most popular type of acupressure is the use of wrist bands that apply pressure to the ventral surface of the wrist. A cross-over pilot study of pediatric cancer patients showed that acupressure is risk-free, feasible and well-accepted [32].

### Mind–body therapies

Many mind–body therapies, such as cognitive distraction, meditation, imagination, creative arts therapy, and hypnosis, are reported to be useful for treating procedure-related anxiety, pain, and distress in pediatric cancer patients. In a small retrospective study, meditation was found to significantly reduce the use of analgesic therapies in children with neuroblastoma on monoclonal antibody therapy [35]. In particular, non-pharmacologic interventions for procedure-associated pain, such as hypnosis, seem beneficial in cancer therapy [36]. A study showed this practice to be particularly useful for pediatric patients (age range 7–14 years) who seem to be more sensitive to hypnosis when used in conjunction with pharmacological therapies [37]. Hypnotherapy refers to many different practices, including relaxation, imagination, and aromatherapy, which are the most used in pediatric cancer patients. Hypnotherapy is the most used mind–body therapy to control nausea and vomiting, the main symptoms associated with sympathetic stimulation, in children with cancer. Through a state of deep relaxation, hypnotherapy helps people easily overcome automatic thoughts such as anticipated nausea and vomiting derived from cancer treatment [37]. A review of all CAM studies for procedure-related distress in pediatric oncology showed that hypnosis is also effective in painful procedures (e.g., lumbar puncture, bone marrow) and for reducing anticipatory anxiety [35].

### Energy therapies

Reiki, therapeutic touch, and healing touch are part of energy therapies. They have not been extensively studied in adults with cancer. Energy healing therapists, also known as biofield therapy practitioners, channel healing energy through the hands into the patient's body to rehabilitate normal energy balance and health. In a review of biofield therapies, it was reported that adults with cancer showed commonly positive effects on reducing pain and psychological distress characterized by anxiety, depression and stress, and improved quality of life [19]. Data on pediatric cancer patients are limited. A small study of healing touch versus a "read/play" control showed a reduction in pain, stress, and fatigue for patients, parents, and caregivers [36]. Energy therapies are well accepted by children and adult cancer patients, as they show no adverse effects. However, conclusive data in favor of their use are lacking [19].

### Selected herbs and biological therapies

Many cancer patients use many herbs and biological therapies [38]. Traditional Chinese Medicine (TCM) is a health management that includes herbal medicines and various mind and body practices to prevent and treat diseases. Usually, TCM practitioners integrate many herbal medicines, and each herbal therapy is planned for each patient. The use of Chinese herbs in the adult and pediatric oncology population is controversial because several reports indicate contamination of the herbs used in therapy with drugs, toxins, or heavy metals. Therefore, their use is currently under monitoring and their content is under evaluation to acquire conclusive data about their safety [37].

Probiotics are biological CAM frequently used in children and adolescents [39]. Probiotics could have a positive effect on allogeneic stem cell transplantation (SCT). Experimentally, significant survival and reduction of acute graft-versus-host disease (aGVHD) before and after transplantation was reported using L. rhamnosus GG in a mouse model of aGVHD [38]. In a small study of chemotherapy-treated children, the use of Bifidobacterium breve strain Yakult reduced fever episodes and improved the presence of anaerobes in the gut microbiota [39]. Glutamine is an essential amino acid that has been used for the prevention of peripheral neuropathy and mucositis. Although the ideal dose and route of administration have not yet been defined, it represents a future option in CAM therapy for adult and pediatric cancer patients. In children undergoing stem cell transplantation, glutamine has helped reduce the duration of fever, and the use of total parental nutrition (TPN) and narcotics has been related to the standard of care protocol [40].

### Use of CAM to relieve pain and anxiety

Pain is a common symptom frequently related to cancer diagnosis procedures and treatment, and it can also result from disease progression, due to obstruction of nerves, tissues, or organs by tumors at any stage of the cancer process [41]. Many studies emphasize the importance of complementary modalities in helping children undergoing cancer treatment in general and especially in painful procedures, including lumbar puncture, bone marrow aspiration, access to implanted ports, and venipuncture [42, 43].

In several studies investigating various painful procedures, mind–body techniques and hypnosis have shown positive results in reducing pain and anxiety. These two noninvasive techniques are reported to help mitigate or control the effects of painful procedures in children during cancer treatment [43, 44].

### The importance of communication in the use of CAM

The most important motivations for the use of CAM have been the goal to improve the patient's overall condition, strengthen the immune system, and reduce the adverse effects of conventional therapy. Parents often do not choose CAM because they lack information about it, are convinced that it is ineffective, and want their children to be stress-free [14].

A recent study emphasizes a lack of communication between pediatric oncologists and their patients [9]. According to this report, 7% of pediatric oncologists never ask their patients an open question about CAM use, while 43% sometimes ask their patients a general question about their use. Pediatric oncologists' questions to their patients about specific CAM therapies depend greatly on the type of therapy. For example, more than one-third of pediatric oncologists routinely ask their patients about the use of dietary supplements, phytotherapy, special diets and vitamins. However, the use of aromatherapy, enzymes, acupuncture, homeopathy, magnets, prayer, chiropractic, guided imagery, martial arts, meditation and yoga is never asked to pediatric patients by their oncology team. The use of vitamins, special diets, nutritional supplements, herbal medicine, and antioxidants is often requested by cancer patients from their physicians. However, they are often discouraged. The reasons why pediatric oncologists do not ask their patients about CAM therapies are lack of time in 49% of cases and lack of knowledge in 47% of cases [9].

In conclusion, the main reasons for cancer patients to use CAM are improvements in physical and psychosocial well-being and increasing hope[45], but also despair, disappointment with some features of standard healthcare, lack of physician–patient relationship, availability, and perceived efficiency [46].

### Complementary therapies in neurological diseases

Use of CAM in neurological diseases has increased in recent years, mainly because they are usually chronic conditions and often associated with various comorbidities. A survey reported that children affected by neurological diseases used CAM more frequently than healthy children (24% vs. 12.6%, respectively) [47].

In Canada, 44% to 76% of children with common neurological conditions reported the use of integrative medicines [48, 49].

CAM therapies mainly used in neurological diseases range from nutritional supplements such as herbs and vitamins to massage and osteopathic manipulation, acupuncture, mind–body therapies and relaxation techniques (Table 3) [47].

**Table 3:** CAM therapies mainly used in neurological diseases

	**Cerebral palsy**	**Epilepsy**	**Headache**	**ASD**	**Tourette syndrome**	**ADHD**
**Acupuncture**	** + **		** + **	** + **	** + **	** + **
**Massages and manipulation**	** + **		** + **	** + **		** + **
**Nutritional supplements**		** + **	** + **	** + **	** + **	** + **
**Music therapy**	** + **					** + **
**Hippotherapy**	** + **			** + **		
**Behavioral treatments**		** + **	** + **		** + **	** + **
**Homeopathy**			** + **			** + **
**Other specific therapies**	Hyperbaric therapy	Yoga	Aromatherapy			

**Fig. 1:**
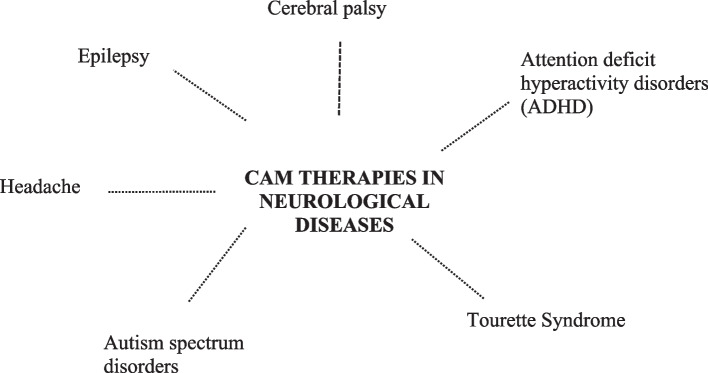
Summary of neurological diseases where CAM therapies are more commonly used

Here in, we analyzed the most common neurological diseases where CAM is used (Fig. 1).

### Cerebral palsy

Cerebral palsy (CP) is a group of disorders characterized by a spectrum of motor and posture impairment caused by non-progressive damage that may happen during prenatal, perinatal, or postnatal stages of the development of the nervous system [50].

The prevalence of CP is estimated to range between 2 and 3 per 1000 live births, representing the leading cause of pediatric disability [51].

Children affected by CP present various neuromotor limitations in their physical activities, leading to a psychological, social and functional impairment [50].

Although neurological injury may happen during each stage of neurological development, perinatal injury accounts for about 90% of all cases and it must be kept in mind in order to prevent and early recognize CP. The principal manifestations of CP are posture, reflexes and muscular impairment, usually associated with sensory problems, coordination imbalance and learning, speech, and cognitive disabilities [52]. Moreover, CP is frequently associated with other neurodevelopmental disorders, particularly attention-deficit hyperactivity disorder (ADHD) represents the most frequently associated comorbidity [53].

Signs and symptoms of CP may be various and range from mild to severe clinical presentations and they may be evaluated according to Gross Motor Function Classification System (GMFCS) [54].

Until now, there is no curative therapy for CP, and the available treatments depend on the specificity of the symptoms. Usually, a multidisciplinary approach is fundamental to reduce the symptoms and improve the quality of life [55].

Many types of therapies are used. The most used conventional treatments include occupational therapies to improve movement and balance impairment, selective dorsal rhizotomy, systemic muscle relaxants, intramuscular on botulinum toxin A to reduce spasticity, and pharmacological therapies to improve neuropsychiatric comorbidities [56]. Among conventional treatments are also included “Vojta therapy” and “Bobath therapy”, which mainly aim to reduce postural and motor imbalance through the reflex mechanism of neurostimulation [57].

On the other hand, as in other chronic neurological conditions, CAM started to be used, especially in the last years. The use of CAM in children affected by CP varies from 27 to 56%, with differences depending on the availability and knowledge of CAM in the different areas [58].

The principal factors associated with CAM use seem to be the type of CP (particularly spastic quadriplegia), the presence of severe motor disabilities and parental use of CAM [59].

Massages are one of the most used CAM practice, particularly in children with severe motor limitations with a prevalence of 15–50% depending on the studies [57, 58]. Hyperbaric oxygen therapy (HBOT) is also a popular therapeutical option; its rationale consists of the reactivation with pressured oxygen of the damaged neuronal cells and the reconstruction of the synapses [60].

A recent systematic review demonstrates high-level evidence that HBOT is ineffective in improving motor and functional performance in children with CP and is associated with some adverse effects such as middle ear barotrauma [61]. Therefore, HBOT is not recommended in CP. Manipulative treatment (OMT), particularly craniosacral treatment and myofascial releases, seems to be useful to improve motor abilities, especially in children with moderate to severe spastic features [62].

Acupuncture use is diffused in children with CP; its benefits are improving the use of legs and arms, warming the extremities, decreasing in the spasm and permitting a better bowel function and sleep [63]. Laser acupuncture is a new type of acupuncture that uses a laser light with low intensity to induce biostimulation of cells and tissue, with similar effects to classic acupuncture but without mechanical effect [64]. Some studies have confirmed the efficacy of laser acupuncture in improving spasticity through biochemical changes in cells and the positive impact on the autonomic system and the neuroprotection role, mediated by increasing the production of neurotropic factors and antioxidant enzymes [65, 66]. Tang et al. have conducted a recent systematic review and metanalysis to evaluate the clinical efficacy of acupuncture with evidence that it has great effect in improving musculoskeletal dysfunction [67].

Music therapy has also been successfully used in CP. Actually, music therapy seems to increase the synchronization between motor and sensorial aspects, facilitating neuroplasticity, and it improves communication and social abilities [68].

Aquatic therapy is a quite recent practice introduced in children with CP. It exercises mechanical (including hydrostatic and hydrodynamic) effects and thermal effects. In fact, aquatic therapy removes gravity, permitting it to perform much more activities than on the land. Lai et al. have conducted a single-bind prospective study analyzing the efficacy of aquatic therapy on sensorial and motor abilities, on all activities of daily living and on the quality of life with the evidence of great benefit on gross motor function and on enjoyment than the in control group [69]. Moreover, equine therapy started to gain visibility due to its mental and physical efficacy in CP. Equine therapy is a holistic practice that provides motor and sensitive stimulation through the horse’s movement and improves social and relational abilities [70]. Although in many countries, equine therapy has many successes in improving CP-associated symptoms, it frequently remains a poorly used practice due to the high costs and parents' doubts regarding its effectiveness and the benefit-risk ratio [71].

Recent studies have analyzed the efficacy of equine therapy with the evidence that it has many positive effects, not only on spasticity, motor and balance control, but also on relational abilities and attention, resulting in an alternative therapy that can be used especially in children with both CP and ADHD [71, 72].

### Headache

Headache is one of the most common disorders, affecting almost 60–75% of children by the age of 15 [73].

Since headache in childhood represents an important physical and psychological disorder with a great impact on the quality of life, multidisciplinary and integrative treatments, based on pharmacological and non-pharmacological approaches, are the best choice to prevent chronicity of symptoms and reduce medication abuse [73].

The use of CAM treatments in childhood headache is increasing, especially in the last years, reaching 76% in some cases [74]. It was observed that parents usually recur to the use of CAM in headache treatment to avoid adverse pharmacological effects and to explore all possible therapeutical options. According to Gaul et al., the use of CAM treatments generally depends on the duration of headache, types of headaches, number of headache episodes and use of CAM for other symptoms [74].

Nutraceuticals are dietary supplements in the form of minerals and vitamins. Riboflavin, coenzyme Q10 and magnesium are the most frequent supplements used in migraine. Riboflavin is involved in cellular function, reduction of oxidative stress and membrane stability [75]. Coenzyme Q10 (CoQ10) acts as an antioxidant agent and maintains an adequate mitochondrial function [76]. Magnesium plays a role in brain cellular homeostasis through interaction with calcium channels, N-methyl-D-aspartate (NMDA) receptors and reducing inflammation. According to the American Academy of Neurology and American Headache Society (AHS/AAN) guidelines, magnesium and riboflavin are considered as Level B of evidence (probably effective for migraine prevention), instead, CoQ10 is considered Level C (possibly effective for migraine prevention) [75].

Interestingly, also vitamin D and melatonin have been used with success to reduce disability and the frequency of headache [77]. Instead, the use of butterbur is no more recommended due to the risk of hepatotoxicity. Danno et al. have demonstrated that also homeopathic medicines, especially *Belladonna, Iris versicolor*, *Ignatia amara*, *Gelsemium* and *Kalium phosphoricum* may be useful to reduce severity and frequency of migraines attacks [78].

Massage therapy may be useful during the attacks. A wide range of massage techniques are available and should be tailored to the type of headache and the patient. The most frequently used massages include deep tissue massage, which acts especially on a chronic tension-type headache; trigger point therapy, based on cycles of pressure and release on a specific point; and Swedish massage, which provides a light-medium pressure [79]. Also, acupuncture practice is widely diffused as CAM in headache. It has been demonstrated its efficacy especially in tension headache and episodic migraine, explaining anti-inflammatory, analgesic, and neurobiological actions. Acupuncture may activate the endorphin-enkephalin system inhibiting pain pathways in the spinal cord and midbrain [80, 81]. Interestingly, Li et al. evaluated the effects of standard acupuncture on right-fronto-parietal networks (RFPN), which are involved in the perception of pain with evidence of reduced activity after 4 weeks of treatment [82]. A Cochrane review demonstrated that acupuncture may be more effective in reducing migraine frequency than prophylactic drug treatment and not receiving acupuncture [83].

Aromatherapy, the medical use of oils derived from plants, is often used in addition to acupuncture due to its anxiolytic properties. It has been observed in a trial that inhaling lavender essential oil for 15 min may significantly reduce the severity of migraine respected the placebo control group [84]. Moreover, the United States Headache Consortium Guidelines recommended with a grade A evidence cognitive behavioral therapy, relaxation techniques and biofeedback in migraine prevention [85]. Cognitive behavioral therapy (CBT) represents one of the leading CAM interventions in children with migraine. The aim of CBT is teaching relaxation, behavioral and cognitive skills to manage attacks and reduce comorbidities and disabilities associated with headache. A recent Cochrane review of psychological therapies for the management of chronic pain evidenced that CBT reduce the number and the severity of attacks [86]. Interestingly, Nahman-Averbuch et al. found that patients with migraine after only 8 sessions of CBT had modifications in connections between prefrontal cortical activity and the amygdala, suggesting a possible organic role in changing the brain area, which regulates emotions and pain regulation [87]. Similarly, relaxation techniques, including muscle relaxation, meditation and diaphragmatic breathing, are mostly used to increase pain control and decrease stress and sympathetic overstimulation [88]. Biofeedback is a self-regulation strategy that aims to regulate autonomic features related to pain and stress, such as breathing, temperature and heartbeat. A metanalysis evaluated the effectiveness of biofeedback on pediatric migraine with the evidence of reduced frequency and duration of migraine attacks and better pain control during the attacks [89].

### Epilepsy

Epilepsy is a chronic condition characterized by a brain predisposition to generate epileptic seizures and it can result from different causes, such as genetic variation or structural brain lesions [90]. In Europe, the estimated number of children and adolescents suffering from epilepsy is 900.000 and worldwide about 10.5 million [91]. The conventional treatment of epilepsy is based on antiepileptic drugs (AEDs), surgery, ketogenic diet and vagus nerve stimulator. AEDs are usually the first-line treatment, but only about two-thirds of patients who use AEDs have optimal control of seizures and AEDs are often associated with adverse effects [92]. Therefore, CAM use in epilepsy has become popular, especially in the last years; however, only a few data are available on the worldwide prevalence of CAM use [93]. A recently published scoping review has shown that CAM usage among children with epilepsy is ranging from 13 to 44% [94].

The main predictor factors of CAM use seem to be the duration and severity of epilepsy, parental use of CAM and incidence of the adverse effect of AEDs [95, 96].

Multivitamins and herbal products are among the most frequently used CAMs and they are generally safe. However, it was reported that some products, such as ginseng and gingko biloba, could trigger seizures due to a pharmacokinetic interaction with AEDs reducing their effectiveness and leading to an exacerbation of seizures [97]. Recent studies evaluated the effectiveness of omega-3 supplementation in patients with epilepsy with the evidence that children supplemented with omega-3 have a significant decrease in the number and severity of seizures. The rationale of omega-3 use seems to be linked to the decrease omega-3 levels observed in patients with epilepsy; actually, omega-3 is involved in the endoplasmic reticulum and myelin function of brain cells, so, a reduction in omega-3 levels may develop seizures [98]. Interestingly, the use of cannabis-related remedies, such as medical marijuana or cannabidiol oil (CBD oil) has increased, especially in refractory epilepsy. The exact antiseizure mechanism is not still known in humans, however, the neuroprotective and anti-inflammatory properties of cannabidiol may influence the protective action of cannabis-related products in epilepsy [99]. A telephone survey conducted among 200 parents/caregivers of children with epilepsy in Pennsylvania reported that 13% of respondents used CAM, particularly, cannabis-related products, with benefits on the frequency of seizures [99].

Also, yoga has been reported in childhood epilepsy [100]. Kanhere et al. have conducted a RCT analyzing the effectiveness of yoga practice as additional therapy to AEDs on epilepsy, and they found that it was associated with seizure freedom respect the control group and, interestingly, with an improvement of EEG with normalization after 6 months of therapy. The probable mechanism of action is a brain desynchronization, increase of gamma-aminobutyric acid (GABA) levels with a shift to a parasympathetic cerebral dominance [101].

Then, it has been reported the use of neurofeedback. Neurofeedback, also known as EEG biofeedback, is a form of conditioning training that aims to regulate brainwaves based on real-time feedback to people about their brainwaves through EEG [102]. Nigro et al. have conducted research regarding the effectiveness of neurofeedback on pediatric epilepsy and they found that still now there is a lack of evidence in the literature to suggest neurofeedback [103]. However, it may be considered as possibly efficacious (level 2) according to association for applied psychophysiology and biofeedback (AAPB) criteria [104].

### Autism spectrum disorders

Autism spectrum disorders (ASD) is a disorder, which usually begins from childhood, characterized by communication and social deficits, associated to restricted interests and anomalous behaviors [105]. The prevalence of ASD is estimated to be under 1%, but it is increasing especially in high-income countries, probably due to increased surveillance [106]. Etiopathogenesis is poorly understood and is thought to be a complex of genetics and environmental factors [107]. The core deficits may range from mild to severe [107]. As well as other neurological chronic conditions, ASD requires long-term and multidisciplinary management [108]. Until now, no specific treatment is known for ASD; nevertheless, many medical and behavioral actions may be useful to improve the relational deficits and the associated comorbidities, such as insomnia, anxiety, hyperactivity and aggression[109]. Based on current healthcare literature, limited conventional treatments are available such as speech and behavioral therapy for communication deficits and occupational therapy to improve motor and sensorial skills. Also, medications such as stimulants, antipsychotics, alpha agonists, antidepressants are often used to improve behavioral defects like aggression, anxiety and agitation without effects on the stereotypy features and social skills. Due to the limited therapeutical options, many parents of children with ASD are interested in CAM. CAM use seems to be particularly associated with ASD severity, parental educational level and length of illness [110].

Nutritional aspects and integration have been extensively studied in the recent years. Particularly, it was found that children with ASD have high levels of harmful amino acids such as tryptophane and phenylalanine and low levels of vitamin B6, vitamin B12, choline and folate which are associated with scarce behavioral and language skills and poor clinical profiles [111]. According to the last evidence, these findings support that vitamin B12 and folate supplementation may be a feasible therapeutical strategy for autistic children [112].

Moreover, some evidence supports a correlation between gut microbiota and ASD. The gut microbiota plays a fundamental role in neuromodulation and inflammation due to its interaction with the enteric nervous system [113]. Autistic children have an altered gut metabolism, with a more absorption of mono-disaccharides in the large intestine, leading to an increase of gastrointestinal fermenting bacteria and a change in a gut microbiota causing osmotic diarrhea. Probiotic administration may modulate gut microbiota and reduce the overproduction of noxious metabolites, promoting the balance between “gut-brain axis” [114]. Do et al. demonstrated that probiotic mixture supplementation *(B. longum and B. bifidum L. acidophilus, L. casei, L. delbrueckii)* improves both gastrointestinal symptoms and communication, social affect score and physical behavior [115]. Similarly, Grossi et al. reported a case of a 12-year-old child with an important cognitive disability that increases social abilities after 4 weeks of probiotic supplementation *(L. paracasei, L. bulgaricus, delbrueckii subsp., S. thermophilus, S. salivarius subsp. B. breve, B. longum, B. infantis, L. acidophilus, L. plantarum)* [116].

Moreover, it was reported a correlation between behavior disorders of ASD and an excess of opioid receptor agonists; particularly, it was supposed that gluten and casein products may increase opioid-like metabolites which reach the blood–brain barrier, leading to an inflammatory response that can negatively influence the neurological development and ASD-correlated symptoms [117, 118]. Knivsberg et al. have studied the efficacy of gluten and casein-free diet, and they suggested avoiding any exclusion diet as a standard treatment [119]. A recent review confirms that there is still insufficient evidence to support a gluten and casein-exclusion diet in all autistic patients [120]. Similarly, omega-3 fatty acids (FAs) supplementation is not effective on ASD-related symptoms and is not recommended [121].

Animal-assisted therapy has also become popular for improving the core deficits of children affected by ASD [122]. Animal-assisted therapy, especially therapeutical horseback riding, may improve communication and social abilities and increase the self-esteem and happiness of children with ASD [122]. Chen et al. have recently conducted a systematic review and metanalysis to evaluate the effectiveness of horseback riding on communicative and social abilities confirming that it can decrease maladaptive symptoms, such as speech impairment. However, they did not find evidence regarding an improvement in stereotypy and hyper sensibilization [123].

Massage therapy has often been performed in children with ASD. Swedish massage, traditional Chinese massage (Tui na) and traditional Indian massage are the most frequently used massages [124]. Some studies reported that massage therapy may increase oxytocin levels and reduce betaendorphin, nitric-oxide and adrenocorticotropin with improvement in the neural circuit, which regards social and communication abilities [125, 126]. On the other hand, Ruan et al. have systematically reviewed the literature-based evidence regarding the effectiveness of massage therapy in children with ASD and reported a lack of data to support massage therapy in children with ASD [127].

Regarding the use of acupuncture, scarce data on its effectiveness in ASD are available. Acupuncture is poorly used in the United States; on the contrary, in China is a widely used treatment, becoming one of the most important CAM used in children with ASD [128]. The main types of acupuncture are scalp acupuncture, electroacupuncture and total body acupuncture [129]. Many studies reported that acupuncture may improve sleep disorders, functional development and control of emotions in children with ASD. Wang et al. conducted a systematic review and metanalysis to evaluate the effectiveness of acupuncture on the core symptoms of ASD. Interestingly, they found that acupuncture may be considered a safe practice and improves attention deficit and affective skills. Nevertheless, they highlight the need for rigorous acupuncture method and prescription, which are still heterogeneous [129].

### Attention deficit hyperactivity disorder

ADHD are common behavioral disorders, with an estimated worldwide prevalence in children of about 5–10% [130].

ADHD is a pervasive disorder that affects sociality and intellectual performance, with a great impact on the quality of life. Clinical manifestations include motor activity, inattention, and impulsivity [130]. Treatment of ADHD is based on a complex of medical, educational, and environmental interventions. The most used and effective medical treatments have been psychostimulants such as primarily methylphenidate [131]. However, although their positive effects, psychostimulants have side effects such as reduction of appetite and sleep disturbance [131]. Therefore, many parents of children with ADHD often recur to the use of CAM treatments. As well as other conditions, high parental education seems to be the most important predictor factor for CAM use [132]. Biochemical treatments, such as herbal treatments, vitamins and nutritional supplements, are considered the most common CAMs administered in ADHD [133]. Herbal remedies, such as kava kava, valerian, chamomile and ginkgo biloba are considered to be useful for their anxiolytic properties to reduce attention deficit and sleep disturbance related to ADHD. They are usually safe, nevertheless, kava kava may interact with alcohol and benzodiazepines increasing their depressant action on the central nervous system [134]. Vitamin and nutritional deficiencies, such as iron deficiency, have been supposed to contribute to hyperactivity and cognitive impairment. Even if they are generally considered safe substances, it must be kept in mind the possible toxic effects of overdoses and the possible contamination with dangerous substances, such as heavy metals [134]. Moreover, until now, in literature strong evidence lacks that integration with vitamins or minerals is effective in ADHD [135].

Mechanical therapies, such as massages and body manipulation, are also frequently used in patients with ADHD. The rationale of massages is to reduce stress, promote relaxation and increase affective and behavioral skills. Ni XQ et al. have conducted a narrative review demonstrating the effectiveness of massages alone or in combination with pharmacotherapies in improving ADHD symptoms [136]. A recent systematic review confirms that massage therapy is efficacious in reducing anxiety and asocial behaviour.

Bioenergetic interventions, such as acupuncture and homeopathy, have also been described in children with ADHD. Ni et al., in a 2015 metanalysis, found that acupuncture was a safe and effective practice, used alone or in combination with medications [137]. Xing et al. have more recently confirmed that acupuncture may be a valid treatment option for ADHD, contributing to relax viscera and promote mind serenity [138].

Homeopathy is a practice based on the “disruption of vital energies” as the origin of illness. Homeopathic treatments are made by the principle that *“like cures like”*; the homeopathic remedies are extreme dilution of plant-derived or animal biomolecules which are target to each specific illness. Only a few data are available in literature; however, according to the recent first systematic review and metanalysis conducted by Gaertner et al., individualized homeopathic remedies may be useful to treat ADHD symptoms [139]. Relaxation techniques, mind–body therapy, biofeedback, hypnosis and meditation have been used to reduce hyperarousal to stress and reduce stress. Particularly, neurofeedback has been reported with good results in controlling impulsivity and aggression. Actually, children with ADHD have an aberrant prevalence of theta waves and low levels of beta waves which conduct to a poor motor, sensorial and impulse control [140].

Moreover, as well as other neurodevelopmental disorders, also in ADHD, the effectiveness of musicotherapy has been evaluated, particularly relating to its effects on cognitive performance which are usually compromised in children with ADHD [141]. Chen et al. analyzed the effect of white noise on cognitive abilities and found that it positively impacts working memory and levels of arousal [142]. Zhu et al. conducted a randomized control trial (RCT) on the effect of musicotherapy associated with cognitive behavioral intervention evaluated by cross-attention tests and found that after 16 weeks of musicotherapy and cognitive behavioral intervention, a dramatic increase in behavior and attention score [143].

### Tourette syndrome

Tourette syndrome (TS) is a neuropsychiatric disorder usually developed in childhood or adolescence and characterized by phonic and motor tics, lasting almost one year. Genetic factors and the increased dopamine circuitry are the most important factors in etiopathogenesis in TS. Since TS is a chronic condition and is frequently associated with many comorbidities, such as ADHD, a multidisciplinary approach is fundamental [144].

Pharmacological intervention plays an important role in the treatment of TS and, although a wide range of therapies are available, only 3 are Food And Drugs (FDA)-approved: aripiprazole (from 6 to 18 years old), pimozide (over 12 years old) and haloperidol (over 3 years old) [145].

Nevertheless, pharmacotherapy has many side effects which may add up over time, and therefore, usually, parents tend to prefer a non-pharmacological approach [146].

Behavioral interventions, including relaxation training, biofeedback, psychoeducation and behavioral rewards, are considered the first-line treatments. Actually, behavioral interventions are useful to reduce the frequency of tics and their severity, although they have a limited effect in case of a severe form of TS [147].

A recent metanalysis of 22 RCTs reported that acupuncture in TS is more effective than medical treatment, considering the reduction of recurrence and severity of the tics the low rate of adverse effects [148]. Interestingly, two metanalysis reported that two Chinese medications (choudongning and ningdong granule) may reduce tics, modulating the dopaminergic pathways [149, 150].

Moreover, it was reported that also other types of supplementations, such as taurine added to tiapride and vitamin D, may improve tics compared to placebo, although more studies are needed to evaluate their effectiveness [151].

### Complementary and alternative medicine (CAM) in liver diseases

Acute and chronic liver diseases have an increasing impact on patient's quality of life. Conventional medical therapies are sometimes of limited efficacy, and patients seek other approaches considered safer and better, due to the possible side effects of the classic drugs. Recent decades have seen an increase in the use of CAM in treating liver disease. Studies in different countries have revealed that 33 to 75% of patients with liver disease use CAM, most frequently in countries where traditional medicine is still widely used. The use of CAM occurs without the advice or knowledge of their physicians, in fact, up to 40% of patients have not disclosed it to their physicians [152, 153]. On the other hand, physicians know little about CAM and mostly discourage its use due the hepatotoxicity [153]. In addition, the risk of toxicity and adverse effects is higher in these patients because of underlying liver disease [154]. Patients with chronic liver disease choose to use CAM in search of benefits in regulating immunity, postponing disease progression, improving quality of life, alleviating adverse effects of conventional therapies or poorly controlled extrahepatic symptoms, and improving survival [153–155]. Phytotherapy has been recommended since 2100 before Christ (BC) [152]. Today it is used in up to 50% of patients with liver disease [153]. Studies have shown that multivitamins and herbal products are mostly used by patients with chronic hepatitis C virus (HCV) infection [156, 157]. Other CAM options would be less used, such as homeopathy by only 4% and acupuncture by 9% of HCV patients in the United States [153]. In addition, liver cancer patients use acupuncture to relieve postoperative pain [158]. CAM most commonly used in liver diseases are reported in Table 4.

**Table 4:** CAM used in liver diseases

Complementary and alternative medicine in liver diseases	Herbs	Silymarin, Glycyrrhizin, *Phyllantus ammarus*
		Blended herbal products: Chinese Traditional Medicine (HM861, CH100) Japanese (TJ-9) Ayurvedic medicine (LIV52)
	Antioxidants	Vitamin E, polyphenols derived from green tea, N-acetyl cysteine, pro-cysteine
	Immune modulators	Polyamines, S-adenosyl-methionine
	Homeopathic drugs	
	Acupuncture	

### Herbal products

Of all the options offered by CAM, the most widely used in liver disease are herbal products. Numerous herbs used by patients with liver disease are considered beneficial, and some have been shown to have effects in experimental studies and animal models. However, the efficacy of these herbal products has been tested in many RCT studies, with some design problems. Most data come from case reports, case series, and uncontrolled studies, many without objective endpoints (histology, viral load, survival) [152, 159].

### Silymarin

Silymarin (Milk thistle) is extracted from Sylibum marianum, a flowering herb related to the daisy and ragweed family, native to Mediterranean countries. It is a complex mixture of polyphenolic molecules. The most important component is silybin (80–90% of the herb's components). Silymarin has been used in Europe since the sixteenth century for liver disease and jaundice and is one of the most extensively tested herbal products in animal models and human studies. The composition and efficacy of silymarin are well documented [159].

Experimental data, in vitro and animal models, have shown that it has many effects that could be considered helpful in liver disease. Silymarin is an important antioxidant and free radical scavenger, prevents glutathione depletion, induces glutathione S-transferase catalase, and prevents free radical formation [160–162].

It has an anti-inflammatory effect by inhibiting nuclear transcription factor (NFκB) and reducing inflammatory cytokines [163]. It also has an antifibrotic role by blocking stellate cell proliferation, regulating TGF beta [164] and reducing collagen accumulation [164]. Silymarin is considered hepatoprotective because it stabilizes the cell membrane of hepatocytes, preventing toxic effects. It prevents liver injury produced by Amanita phalloides, carbon tetrachloride (CCl4) and paracetamol. The maximum effect was demonstrated in the case of pretreatment, but also after exposure of the Balb/c mouse animal model to the toxic substances to induce experimental liver injury [153].

Regarding human studies, the first RCT study, although having several design elements, showed important effects in terms of improved survival in patients with mild cirrhosis (Child A) and alcohol-induced liver injury. No side effects were reported [165]. Other studies on alcohol-related liver disease have not made a clear conclusion on the usefulness of silymarin [165, 166].

In studies that included patients with chronic viral hepatitis, some decrease in alanine-aminotransferase (ALT) level was reported, but no change in HCV viral load [166, 167]. Other studies have shown no change [168].

Regarding acute hepatitis, studies have shown a shorter duration of hospitalization and an improvement in liver enzyme levels [152] but there have been studies with contradictory results [169]. There have been no significant improvements in patients with primary biliary cirrhosis [170]. A major problem with studies using silymarin is the lack of a reliable formulation for studies and a large variability in peak drug levels. On the other hand, silymarin in an intravenous form is recommended to treat mushroom poisoning [152, 171] as it demonstrates a protective role against various drugs and toxins.

A recent systematic review including 17 studies and a meta-analysis including 6 studies on the role of silymarin in patients with nonalcoholic fatty liver disease (NAFLD) showed that there was an association with reduced liver enzyme levels, but without clinical significance, and it was well tolerated and without adverse effects [172].

### Glycyrrhizin

Glycyrrhizin (licorice root extract) is an aqueous extract of licorice root, Glycyrrhiza glabra, native to the Mediterranean region and the Middle East [152, 153]. It is recommended to treat cough, bronchitis, gastritis and liver diseases [173]. The main constituents are glycyrrhetinic acid, flavonoids, isoflavonides, hydroxycoumarins, triterpenoids, and polysterols [152]. Glycyrrhizin is a component of many herbal medicines [152].

Experimental studies, in vitro and animal models, have shown that glycyrrhizin inhibits the activity of 11-beta-hydroxysteroid dehydrogenase, inhibits the production of prostaglandins (PG) E2 by macrophages, and modifies the metabolism of arachidonic acid [174]. It has antioxidant activity by inducing the activity of glutathione-S-transferase and catalase and decreasing the formation of oxidative products of polymorphonuclear cells. It has proven antifibrotic activity, blocking the activation and action of NFkB [175] and inhibiting tumor necrosis factor (TNF) [176].

In clinical studies, a possible benefit in HCV infection was considered, with an improvement in liver enzymes, but without a demonstrated change in viral load. The same results were obtained in studies that included patients with hepatis B virus (HBV) infection [152].

Glycyrrhizin includes a beta-sitosteroid, which may have glucocorticoid and mineralocorticoid activity. Side effects due to mineralocorticoid activity have been reported in clinical trials: increased severity of cirrhosis, fluid retention, and hyperkalemia. Because of these effects, glycyrrhizin should be avoided in patients with cirrhosis [153].

### Cucurmin

Curcumin is the principal curcuminoid of turmeric (Curcuma longa), a member of the ginger family, Zingiberaceae. It is a natural polyphenolic compound used in liver diseases for its antioxidant and anti-inflammatory effects, and it was recommended as early as 250 B.C. in Ayurvedic medicine to counteract food poisoning [177]. Numerous reports and clinical studies support its beneficial role in NAFLD, autoimmune hepatitis, HCV, and hepatocarcinoma (HCC) [178, 179]. In vitro studies have demonstrated its action as a free radical scavenger, reducing lipid peroxidation, increasing the expression of glutathione-S-transferase, glutathione reductase and peroxidase, superoxide dismutase and catalase, reducing ossid nitric (NO) production and inhibiting Reacting Oxygen Species (ROS) formation [180, 181]. It also suppresses NFκB and intervenes in other steps of fibrogenesis [179]. In a systematic review and meta-analysis on the role of curcumin supplementation in patients with NAFLD, including 9 RCTs, the favorable effect on metabolic markers and anthropometric parameters was supported [182].

In addition to unique herbal products, blended herbal formulations or extracts are used in different cultures. Traditional Chinese, Japanese, Ayurvedic, and other medicines use herbs in blended products, and practitioners find it challenging to analyze each component separately, as efficacy may be lost [183].

Traditional Chinese medicine includes several practices, such as acupuncture, herbal therapies, moxibustion (dermal therapy against irritation), massage, and exercise therapy (Qi Gong) [153]. Of the more than 100,000 known herbal therapies in traditional Chinese medicine, about 76 mixtures are used in liver diseases [153].

*Plantago asiatica* seeds have a hepatoprotective role and lower toxicity due to its active compound, aucubin. In vitro and animal model studies have shown that aucubin inhibits HBV replication, but human studies have shown that this is only a temporary effect [184]. It is known to modulate cytokine release through the NFkB pathway [185] with an antifibrotic and anti-inflammatory effect.

Herbal Medicine 861 (HM861) is composed of 10 herbs, including Salvia miltiorrhiza, Astragalus membranaceus, and Spatholobus suberectus [186]. In vitro studies have shown that HM861 inhibits proliferation and induces apoptosis of hepatic stellate cells (HSCs) [187] and corrects the imbalance between extracellular matrix synthesis and degradation. Human studies have reported antifibrotic activity in patients with HBV infection (improvement in transaminase level, spleen size, portal pressure, and serum level of procollagen peptide and laminin, with a demonstrated histologic reduction in fibrosis and inflammatory infiltrates) [153].

CH-100 is composed of 19 herbs. In animal studies, it protects against ConA-mediated hepatitis or CCl4 liver injury in rats [188]. Human studies showed a decrease in transaminase levels, but there was no change in viral load in HCV patients [189].

Traditional Japanese medicine with Kampo extracts also originates from traditional Chinese medicine. TJ-9 (Sho-saiko-to) is a dried decoction of 7 herbs (scutellaria root, glycyrrhizin, bupleurum, ginseng, pinella tuber, jujube fruit, ginger rhizome) [152]. Studies in vitro and animal models have shown that TJ-9 reduces fibrosis through inhibition of HSC activation, decreases hepatic collagen levels, alpha-smooth muscle actin and collagen type 1 expression [153, 190] and also inhibits lipid peroxidation (due to scutellaria, baicalin, and biacal alkaloids) [152]. In human studies, along with conventional treatment in HBV patients, TJ-9 improves liver function, with decreased development of HCC, increased survival, and increased TNF-alpha and granulocyte colony-stimulating factor. No adverse effects have been found, but it may produce interstitial pneumonia when used together with interferon or even hepatotoxicity (without knowing the component that induces it) [152].

Ayurvedic medicine, traditional Indian medicine with a history of more than 5,000 years, considers the liver as part of the harmony of the whole body and includes diet and meditation in addition to herbs [153]. LIV 52 is an herbal formulation that includes Capparis spinosa (capers), Cichorium intybus (wild chicory), Terminalia arjuna (ajuna), Solanum nigrum (black nightshade), Cassia occidentalis (Kasamarda), Achillea millefolium (yarrow), Mandur bhasma, Tamarx gallica (tatarisk). In animal models, LIV 52 protects against liver injury produced by CCl4 or alcohol. It improves liver function in acute hepatitis, but studies in alcoholic hepatitis have shown worse survival in patients with severe cirrhosis (Child C) [191].

Phyllanthus amarus, also known as bahupatra, contains philanthines, hypophyllanthines, and polyphenols. There are conflicting reports on its possible role in chronic HBV infection. In vitro studies have shown that it has a role in HBV down-regulation [192]. One article, reporting a group of RCT studies, showed a beneficial effect in HBsAg elimination [193], but other studies have failed to demonstrate this [194].

Drugs targeting immune dysregulation as a component of liver disease pathogenesis could effectively treat these patients. Antioxidants (vitamin E) or glutathione prodrugs (N-acetylcysteine, procysteine) have been shown to inhibit TNF, IL8, or IL6 produced by PMNs, monocytes, and Kupfer cells and could be effective in alcohol-induced liver disease [152]. Polyphenols derived from green tea (Camellia sinensis) have antioxidant and anti-cytokine roles and may be helpful in autoimmune liver disease [153, 195]. The use of polyamines and S-adenosyl-methionine (known as immunonutrition) protects against TNF hepatotoxicity, in vitro and animal models. Their use has also been evaluated in human studies of alcohol-induced liver disease [196, 197]. Still, a Cochrane systematic review failed to demonstrate a benefit Zinc, selenium, and vitamin C may regulate liver tissue repair by acting on DNA metabolism [198] and, together with vitamin E, may play a role in improving fibrosis in nonalcoholic steatohepatitis [195].

### Herbal toxicity

It is important for physicians and their patients to know the risk of hepatotoxicity from various herbal products. There are many reports on the possible toxic reactions of CAM. Because patients keep CAM use to themselves, diagnosis is difficult, and physicians should openly discuss all products used with their patients [153]. Although it is challenging to evaluate reports of hepatotoxicity because of the lack of manufacturing standards, or possible experimental studies without feedback in clinical life, here we present some agents with well-documented evidence of hepatotoxicity (Table 5). In addition to direct hepatotoxicity, possible interactions between conventional drugs and herbal products are to be considered [153]. Herbs may be safe when used alone, while adverse reactions increase when used together with conventional drugs [197].

**Table 5:** Herbal products with well-documented hepatotoxicity [152, 199]

**Type of liver disease or injury and the causative herbal product**
**Veno-occlusive disease**: pyrrolizidine alkaloids (Senecio longilobus, Heliotropium europaenum, Crotalaria species, Symphytum officinale, Grodolobo herbal tea)
**Zone 3 necrosis, cirrhosis**: chapparal leaf (Larrea tridenta), germander (Teucrium chamaedrys), pennyroyal (squawmit oil, Mentha pulgeium)
**Acute liver injury**: Jin Bu Huan, tradititional Chinese herbs, Kava, Kombucha mushroom (tea)
**Microvesicular steatosis**: margosa oil **Microvesicular steatosis**: margosa oil

In summary, there is an increase in the use of CAM in patients with liver disease, and generally without physicians' knowledge. Therefore, physicians should openly and routinely discuss the use of CAM as part of their patients' medication use history. In addition, they should be aware of the efficacy and safety of different alternative methods. By far, the most widely used CAM in liver disease patients are herbal products, which have shown protective actions in vitro or animal model studies, but whose efficacy has not been demonstrated in adequately conducted RCT studies. A reliable and standardized preparation of these products is needed to conduct well-designed studies demonstrating their efficacy and safety, as hepatotoxicity can be a severe effect of using herbal products.

## Conclusion

The use of CAM is increasingly becoming part of the treatment of some serious diseases in pediatric patients. CAM is used to improve the success of conventional therapies, but also to alleviate the pain, discomfort, and suffering resulting from the diseases and their treatment, which are often associated with a significant burden of adverse effects. The use of CAM in children also has a major impact on parents' ability to cope with their children's serious illnesses, as it can provide relief from symptoms that most affect their quality of life.

This review has some limitations. Firstly, its narrative nature, that is mainly descriptive, has no systematic and formal approach; therefore, it can include an element of selection bias. The literature on the use of CAM in oncological, neurological and liver diseases in children is abundant and growing; however, knowledge of their efficacy and safety is still incomplete, especially for some products. As a second limitation, in this review we focused only on these conditions; however, use of CAM is increasingly becoming common in other conditions and diseases, too. Therefore, more high-quality studies are still needed to provide advice on product types and indications for use. Finally, a better understanding by physicians of the opportunities offered by the use of CAM is advisable.

## Data Availability

Not applicable.

## Abbreviations

CAM: Complementary and alternative medicine
NIH: National Institutes of Health
NCCIH: National Center for Complementary and Integrative
HDS: Health herbal dietary supplements
NCCAM: National Center for Complementary and Alternative
BNT: Bone narrow transplantation
TCM: Traditional Chinese Medicine
aGVHD: Acute graft-versus-host disease
CP: Cerebral palsy
ADHD: Attention-deficit hyperactivity disorder
GMFCS: Gross Motor Function Classification System
HBOT: Hyperbaric oxygen Therapy
OMT: Manipulative Treatment
CoQ10: Coenzyme Q10
NMDA: N-methyl-D-aspartate
AHS/AAN: American Academy of Neurology and American Headache Society
RFPN: Right-fronto-parietal networks
CBT: Cognitive Behavioral therapy
AEDs: Anti-epileptic drugs
CBD oil: Cannabidiol Oil
GABA: Gamma-Aminobutyric Acid
AAPB: Association for applied psychophysiology and biofeedback
ASD: Autism Spectrum Disorder
FAs: Omega 3 Fatty Acids
RCT: Randomized Control Trial
TS: Tourette Syndrome
FDA: Food And Drug Administration
BC: Before Christ
HCV: Hepatis C Virus
NFκB: Inhibiting Nuclear Transcription Factor
CCl4: Carbon Tetrachloride
ALT: Alanine-Aminotransferase
NAFLD: Nonalcoholic Fatty Liver Disease
PG: Prostaglandins
TNF: Tumor Necrosis Factor
HBV: Hepatitis B Virus
HCC: Hepatocarcinoma
HSCs: Hepatic Stellate Cells
NO: Ossid Nitric
ROS: Reacting oxygen species

## References

[1] Spiegel D, Stroud P, Fyfe A (1998). Complementary medicine. West J Med.

[2] Pirson L, Lüer SC, Diezi M, Kroiss S, Brazzola P, Schilling FH, et al. Pediatric oncologists’ perspectives on the use of complementary medicine in pediatric cancer patients in Switzerland: A national survey‐based cross‐sectional study. Cancer Reports. 2023;6. Available from: 10.1002/cnr2.1649. [cited 2023 Feb 19]. 10.1002/cnr2.1649 PMC9875643 35699504

[3] Cochrane. Complementary Medicine. Available from: https://cam.cochrane.org/about-us/our-partners. [cited 2023 Feb 23].

[4] Zhang CS, Tan HY, Zhang GS, Zhang AL, Xue CC, Xie YM. Placebo Devices as Effective Control Methods in Acupuncture Clinical Trials: A Systematic Review. Weng X, editor. PLoS ONE. 2015;10:e0140825. 10.1371/journal.pone.0140825 PMC4633221 26536619

[5] Lombardi N, Crescioli G, Bettiol A, Menniti-Ippolito F, Maggini V, Gallo E (2019). Safety of complementary and alternative medicine in children: A 16-years retrospective analysis of the Italian Phytovigilance system database. Phytomedicine.

[6] Lewith Kenyon J, Lewis P. Complementary medicine: an integrated approach. Oxford: Oxford University Press, 1996 (Oxford General Practice Series).;

[7] National Center for Complementary and Alternative Medicine, National Institutes of Health, and US Department of Health and Human Services, M-BM. What Is Complementary and Alternative Medicine. 2012. Available from: http://nccam.nih.gov/health/whatiscam/.

[8] Renee A. Bellanger, Christina M. Seege. Complementary and alternative medicine. Sidhartha D. Ray; 2020.

[9] Roth M, Lin J, Kim M, Moody K (2009). Pediatric Oncologists’ Views Toward the Use of Complementary and Alternative Medicine in Children With Cancer. J Pediatr Hematol Oncol.

[10] Magi T, Kuehni CE, Torchetti L, Wengenroth L, Lüer S, Frei-Erb M. Use of Complementary and Alternative Medicine in Children with Cancer: A Study at a Swiss University Hospital. Sethi G, editor. PLoS ONE. 2015;10:e0145787. 10.1371/journal.pone.0145787 PMC4687920 26694320

[11] Bishop FL, Prescott P, Chan YK, Saville J, von Elm E, Lewith GT (2010). Prevalence of complementary medicine use in pediatric cancer: a systematic review. Pediatrics.

[12] Horneber M, Bueschel G, Dennert G, Less D, Ritter E, Zwahlen M (2012). How Many Cancer Patients Use Complementary and Alternative Medicine: A Systematic Review and Metaanalysis. Integr Cancer Ther.

[13] Kranjcec I, Abdovic S, Buljan D, Matijasic N, Slukan M, Stepan J. Complementary Medicine Practice and Use of Dietary Supplements in Pediatric Cancer Patients in Croatia. Cureus. 2022; Available from: https://www.cureus.com/articles/112632-complementary-medicine-practice-and-use-of-dietary-supplements-in-pediatric-cancer-patients-in-croatia. [cited 2023 Feb 19]. 10.7759/cureus.30246 PMC9652699 36381903

[14] Laengler A, Spix C, Seifert G, Gottschling S, Graf N, Kaatsch P (2008). Complementary and alternative treatment methods in children with cancer: A population-based retrospective survey on the prevalence of use in Germany. Eur J Cancer.

[15] Jacobs S (2014). Integrative Therapy Use for Management of Side Effects and Toxicities Experienced by Pediatric Oncology Patients. Children.

[16] Melchionda F, Spreafico F, Ciceri S, Lima M, Collini P, Pession A (2013). A novel WT1 mutation in familial wilms tumor: Novel WT1 Mutation in Familial Wilms Tumor. Pediatr Blood Cancer.

[17] Gomez-Martinez R, Tlacuilo-Parra A, Garibaldi-Covarrubias R (2007). Use of complementary and alternative medicine in children with cancer in Occidental. Mexico Pediatr Blood Cancer.

[18] Poder TG, Lemieux R (2013). How Effective Are Spiritual Care and Body Manipulation Therapies in Pediatric Oncology? A Systematic Review of the Literature. GJHS.

[19] Thrane S (2013). Effectiveness of Integrative Modalities for Pain and Anxiety in Children and Adolescents With Cancer: A Systematic Review. J Pediatr Oncol Nurs.

[20] Jane S-W, Wilkie DJ, Gallucci BB, Beaton RD (2008). Systematic Review of Massage Intervention for Adult Patients With Cancer: A Methodological Perspective. Cancer Nurs.

[21] Mehling WE, Lown EA, Dvorak CC, Cowan MJ, Horn BN, Dunn EA (2012). Hematopoietic Cell Transplant and Use of Massage for Improved Symptom Management: Results from a Pilot Randomized Control Trial. Evidence-Based Complement Alternat Med.

[22] Ernst E (2009). Massage therapy for cancer palliation and supportive care: a systematic review of randomised clinical trials. Support Care Cancer.

[23] Myers CD, Walton T, Small BJ. The value of massage therapy in cancer care. Hematol Oncol Clin North Am. 2008;22:649–60, viii. 10.1016/j.hoc.2008.04.003 18638693

[24] Ackerman SL, Lown EA, Dvorak CC, Dunn EA, Abrams DI, Horn BN (2012). Massage for Children Undergoing Hematopoietic Cell Transplantation: A Qualitative Report. Evidence-Based Complement Alternat Med.

[25] Russell NC, Sumler S-S, Beinhorn CM, Frenkel MA (2008). Role of Massage Therapy in Cancer Care. J Alternat Complement Med.

[26] Kirkwood G (2005). Yoga for anxiety: a systematic review of the research evidence * Commentary. Br J Sports Med.

[27] Pilkington K, Kirkwood G, Rampes H, Richardson J (2005). Yoga for depression: The research evidence. J Affect Disord.

[28] Geyer R, Lyons A, Amazeen L, Alishio L, Cooks L (2011). Feasibility Study: The Effect of Therapeutic Yoga on Quality of Life in Children Hospitalized With Cancer. Pediatr Phys Ther.

[29] Thygeson MV, Hooke MC, Clapsaddle J, Robbins A, Moquist K (2010). Peaceful Play Yoga: Serenity and Balance for Children With Cancer and Their Parents. J Pediatr Oncol Nurs.

[30] Fukuhara JS, O’Haver J, Proudfoot JA, Spies JM, Kuo DJ (2020). Yoga as a Complementary and Alternative Therapy in Children with Hematologic and Oncologic Disease. J Pediatr Oncol Nurs.

[31] Manne S, Mee L, Bartell A, Sands S, Kashy DA (2016). A randomized clinical trial of a parent-focused social-cognitive processing intervention for caregivers of children undergoing hematopoetic stem cell transplantation. J Consult Clin Psychol.

[32] Wu S, Sapru A, Stewart MA, Milet MJ, Hudes M, Livermore LF (2009). Using acupuncture for acute pain in hospitalized children. Pediatr Crit Care Med.

[33] Ladas EJ, Rooney D, Taromina K, Ndao DH, Kelly KM (2010). The safety of acupuncture in children and adolescents with cancer therapy-related thrombocytopenia. Support Care Cancer.

[34] Jones E, Isom S, Kemper KJ, McLean TW (2008). Acupressure for chemotherapy-associated nausea and vomiting in children. J Soc Integr Oncol.

[35] Kanitz JL, Camus MEM, Seifert G (2013). Keeping the balance – an overview of mind–body therapies in pediatric oncology. Complement Ther Med.

[36] Wong J, Ghiasuddin A, Kimata C, Patelesio B, Siu A (2013). The Impact of Healing Touch on Pediatric Oncology Patients. Integr Cancer Ther.

[37] Marshall AC. Traditional Chinese Medicine and Clinical Pharmacology. In: Hock FJ, Gralinski MR, editors. Drug Discovery and Evaluation: Methods in Clinical Pharmacology. Cham: Springer International Publishing; 2020. p. 455–82. Available from: 10.1007/978-3-319-68864-0_60. [cited 2023 Feb 19].

[38] Gerbitz A, Schultz M, Wilke A, Linde H-J, Schölmerich J, Andreesen R (2004). Probiotic effects on experimental graft-versus-host disease: let them eat yogurt. Blood.

[39] Wada M, Nagata S, Saito M, Shimizu T, Yamashiro Y, Matsuki T (2010). Effects of the enteral administration of Bifidobacterium breve on patients undergoing chemotherapy for pediatric malignancies. Support Care Cancer.

[40] Stachowicz-Stencel T, Synakiewicz A (2012). Glutamine as a supplemental treatment in pediatric and adult oncology patients. Expert Opin Investig Drugs.

[41] Wee B, Hillier R (2008). Pain control. Medicine.

[42] Jay S, Elliott CH, Fitzgibbons I, Woody P, Siegel S (1995). A comparative study of cognitive behavior therapy versus general anesthesia for painful medical procedures in children. Pain.

[43] Liossi C, White P, Hatira P (2006). Randomized clinical trial of local anesthetic versus a combination of local anesthetic with self-hypnosis in the management of pediatric procedure-related pain. Health Psychol.

[44] Smith J.T., Barabasz A. Comparison of hypnosis and distraction in severely ill children undergoing painful medical procedures J Couns Psychol 43(2):187-95.

[45] Mora DC, Overvåg G, Jong MC, Kristoffersen AE, Stavleu DC, Liu J (2022). Complementary and alternative medicine modalities used to treat adverse effects of anti-cancer treatment among children and young adults: a systematic review and meta-analysis of randomized controlled trials. BMC Complement Med Ther.

[46] Swisher EM, Cohn DE, Goff BA, Parham J, Herzog TJ, Rader JS (2002). Use of Complementary and Alternative Medicine among Women with Gynecologic Cancers. Gynecol Oncol.

[47] Treat L, Liesinger J, Ziegenfuss JY, Humeniuk K, Prasad K, Tilburt JC (2014). Patterns of Complementary and Alternative Medicine Use in Children with Common Neurological Conditions. Glob Adv Health Med.

[48] Soo I, Mah JK, Barlow K, Hamiwka L, Wirrell E (2005). Use of Complementary and Alternative Medical Therapies in a Pediatric Neurology Clinic. Can j neurol sci.

[49] Galicia-Connolly E, Adams D, Bateman J, Dagenais S, Clifford T, Baydala L, et al. CAM Use in Pediatric Neurology: An Exploration of Concurrent Use with Conventional Medicine. Lafrenie R, editor. PLoS ONE. 2014;9:e94078. 10.1371/journal.pone.0094078 PMC3988088 24736474

[50] Pérez Martínez R (2014). Efectos de la Hipoterapia en Personas con Parálisis Cerebral: Una Revisión Sistemática.

[51] Koman LA, Smith BP, Shilt JS (2004). Cerebral palsy. Lancet.

[52] Villegas Guerrero, M.A. Aplicación de la Hipoterapia en niños/as con Parálisis Cerebral Infantil de 3 a 12 Años, Para Mejorar sus Funciones Neuromusculoesqueléticas y Relacionadas con el Movimiento, en la Unidad de Equitación y Remonta de la Policía Nacional por el Período Novie. Universidad Central del Ecuador: Quito, Ecuador. 2018;

[53] Bjorgaas HM, Elgen I, Boe T, Hysing M (2013). Mental health in children with cerebral palsy: does screening capture the complexity?. ScientificWorldJournal.

[54] O’Shea TM (2008). Diagnosis, treatment, and prevention of cerebral palsy. Clin Obstet Gynecol.

[55] Novak I (2014). Evidence-based diagnosis, health care, and rehabilitation for children with cerebral palsy. J Child Neurol.

[56] Majnemer A, Shikako-Thomas K, Shevell MI, Poulin C, Lach L, Schmitz N (2013). Pursuit of Complementary and Alternative Medicine Treatments in Adolescents With Cerebral Palsy. J Child Neurol.

[57] Oskoui M, Ng P, Zaman M, Buckley D, Kirton A, van Rensburg E (2021). Complementary and Alternative Therapy Use in Children with Cerebral Palsy. Can J Neurol Sci.

[58] Samdup DZ, Smith RG, Il SS (2006). The use of complementary and alternative medicine in children with chronic medical conditions. Am J Phys Med Rehabil.

[59] Vitrikas K, Dalton H, Breish D (2020). Cerebral Palsy: An Overview. Am Fam Physician.

[60] Marshall RS (2004). The functional relevance of cerebral hemodynamics: why blood flow matters to the injured and recovering brain. Curr Opin Neurol.

[61] Laureau J, Pons C, Letellier G, Gross R (2022). Hyperbaric oxygen in children with cerebral palsy: A systematic review of effectiveness and safety. PLoS ONE.

[62] Duncan B, McDonough-Means S, Worden K, Schnyer R, Andrews J, Meaney FJ (2008). Effectiveness of osteopathy in the cranial field and myofascial release versus acupuncture as complementary treatment for children with spastic cerebral palsy: a pilot study. J Am Osteopath Assoc.

[63] Svedberg LE, Nordahl UE, Lundeberg TC (2001). Effects of acupuncture on skin temperature in children with neurological disorders and cold feet: an exploratory study. Complement Ther Med.

[64] Putri DE, Srilestari A, Abdurrohim K, Mangunatmadja I, Wahyuni LK (2020). The Effect of Laser Acupuncture on Spasticity in Children with Spastic Cerebral Palsy. J Acupunct Meridian Stud.

[65] Litscher G, Opitz G (2012). Technical Parameters for Laser Acupuncture to Elicit Peripheral and Central Effects: State-of-the-Art and Short Guidelines Based on Results from the Medical University of Graz, the German Academy of Acupuncture, and the Scientific Literature. Evid Based Complement Alternat Med.

[66] Qi Y-C, Xiao X-J, Duan R-S, Yue Y-H, Zhang X-L, Li J-T (2014). Effect of acupuncture on inflammatory cytokines expression of spastic cerebral palsy rats. Asian Pac J Trop Med.

[67] Tang Y, Cao Z, Xia Y, Liu Y, Zhang W (2021). Effectiveness and safety of pure acupuncture and moxibustion in the treatment of children with cerebral palsy: A protocol for systematic review and meta analysis. Medicine.

[68] Marrades-Caballero E, Santonja-Medina CS, Sanz-Mengibar JM, Santonja-Medina F (2018). Neurologic music therapy in upper-limb rehabilitation in children with severe bilateral cerebral palsy: a randomized controlled trial. Eur J Phys Rehabil Med.

[69] Lai C-J, Liu W-Y, Yang T-F, Chen C-L, Wu C-Y, Chan R-C (2015). Pediatric aquatic therapy on motor function and enjoyment in children diagnosed with cerebral palsy of various motor severities. J Child Neurol.

[70] Ahn B, Joung Y-S, Kwon J-Y, Lee DI, Oh S, Kim B-U (2021). Effects of equine-assisted activities on attention and quality of life in children with cerebral palsy in a randomized trial: examining the comorbidity with attention-deficit/hyperactivity disorder. BMC Pediatr.

[71] Menor-Rodríguez MJ, Sevilla Martín M, Sánchez-García JC, Montiel-Troya M, Cortés-Martín J, Rodríguez-Blanque R (2021). Role and Effects of Hippotherapy in the Treatment of Children with Cerebral Palsy: A Systematic Review of the Literature. J Clin Med.

[72] Jiménez de la Fuente, A. Efectos de las terapias ecuestres en personas con parálisis cerebral. Rev. Española Discapac. 2017;5, 171–184.

[73] Steiner TJ, Stovner LJ, Katsarava Z, Lainez JM, Lampl C, Lantéri-Minet M (2014). The impact of headache in Europe: principal results of the Eurolight project. J Headache Pain.

[74] Gaul C, Eismann R, Schmidt T, May A, Leinisch E, Wieser T (2009). Use of complementary and alternative medicine in patients suffering from primary headache disorders. Cephalalgia.

[75] Loder E, Burch R, Rizzoli P (2012). The 2012 AHS/AAN guidelines for prevention of episodic migraine: a summary and comparison with other recent clinical practice guidelines. Headache.

[76] Littarru GP, Tiano L (2010). Clinical aspects of coenzyme Q10: an update. Nutrition.

[77] Fallah R, Shoroki FF, Ferdosian F (2015). Safety and efficacy of melatonin in pediatric migraine prophylaxis. Curr Drug Saf.

[78] Danno K, Colas A, Masson J-L, Bordet M-F (2013). Homeopathic treatment of migraine in children: results of a prospective, multicenter, observational study. J Altern Complement Med.

[79] Holland S, Silberstein SD, Freitag F, Dodick DW, Argoff C, Ashman E (2012). Evidence-based guideline update: NSAIDs and other complementary treatments for episodic migraine prevention in adults: report of the Quality Standards Subcommittee of the American Academy of Neurology and the American Headache Society. Neurology.

[80] Langevin HM, Yandow JA (2002). Relationship of acupuncture points and meridians to connective tissue planes. Anat Rec.

[81] Langevin HM, Sherman KJ (2007). Pathophysiological model for chronic low back pain integrating connective tissue and nervous system mechanisms. Med Hypotheses.

[82] Li K, Zhang Y, Ning Y, Zhang H, Liu H, Fu C (2015). The effects of acupuncture treatment on the right frontoparietal network in migraine without aura patients. J Headache Pain.

[83] Linde K, Allais G, Brinkhaus B, Fei Y, Mehring M, Vertosick EA, et al. Acupuncture for the prevention of episodic migraine. Cochrane Database Syst Rev. 2016;2016:CD001218. 10.1002/14651858.CD001218.pub3 PMC4977344 27351677

[84] Sasannejad P, Saeedi M, Shoeibi A, Gorji A, Abbasi M, Foroughipour M (2012). Lavender essential oil in the treatment of migraine headache: a placebo-controlled clinical trial. Eur Neurol.

[85] Silberstein SD (2000). Practice parameter: evidence-based guidelines for migraine headache (an evidence-based review): report of the Quality Standards Subcommittee of the American Academy of Neurology. Neurology.

[86] Fisher E, Law E, Dudeney J, Palermo TM, Stewart G, Eccleston C. Psychological therapies for the management of chronic and recurrent pain in children and adolescents. Cochrane Database Syst Rev. 2018;9:CD003968. 10.1002/14651858.CD003968.pub5 PMC6257251 30270423

[87] Nahman-Averbuch H, Schneider VJ, Chamberlin LA, Kroon Van Diest AM, Peugh JL, Lee GR, et al. Alterations in Brain Function After Cognitive Behavioral Therapy for Migraine in Children and Adolescents. Headache. 2020;60:1165–82. 10.1111/head.13814 32323877

[88] Meyer B, Keller A, Wöhlbier H-G, Overath CH, Müller B, Kropp P (2016). Progressive muscle relaxation reduces migraine frequency and normalizes amplitudes of contingent negative variation (CNV). J Headache Pain.

[89] Stubberud A, Varkey E, McCrory DC, Pedersen SA, Linde M (2016). Biofeedback as Prophylaxis for Pediatric Migraine: A Meta-analysis. Pediatrics.

[90] Fisher RS, Acevedo C, Arzimanoglou A, Bogacz A, Cross JH, Elger CE (2014). ILAE Official Report: A practical clinical definition of epilepsy. Epilepsia.

[91] Kuchenbuch M, Chemaly N, Henniene KM, Kaminska A, Chiron C, Nabbout R (2018). Off-label use and manipulations of antiepileptic drugs in children: Analysis of the outpatient prescriptions in a tertiary center. Epilepsy Behav.

[92] Tolaymat A, Nayak A, Geyer JD, Geyer SK, Carney PR (2015). Diagnosis and Management of Childhood Epilepsy. Curr Probl Pediatr Adolesc Health Care.

[93] Kwan P, Arzimanoglou A, Berg AT, Brodie MJ, Allen Hauser W, Mathern G (2009). Definition of drug resistant epilepsy: Consensus proposal by the ad hoc Task Force of the ILAE Commission on Therapeutic Strategies: Definition of Drug Resistant Epilepsy. Epilepsia.

[94] Zhu Z, Dluzynski D, Hammad N, Pugalenthi D, Walser SA, Mittal R (2023). Use of Integrative, Complementary, and Alternative Medicine in Children with Epilepsy: A Global Scoping Review. Children.

[95] Goker Z, Serin HM, Hesapcioglu S, Cakir M, Sonmez FM (2012). Complementary and alternative medicine use in Turkish children with epilepsy. Complement Ther Med.

[96] Tonekaboni SH, Jafari Naeini S, Khajeh A, Yaghini O, Ghazavi A, Abdollah GF (2014). Use of complementary and alternative medicine for epileptic children in tehran: a cross-sectional study (2009–2011). Iran J Child Neurol.

[97] Samuels N, Finkelstein Y, Singer SR, Oberbaum M (2008). Herbal medicine and epilepsy: proconvulsive effects and interactions with antiepileptic drugs. Epilepsia.

[98] Sohouli MH, Razmpoosh E, Zarrati M, Jaberzadeh S (2022). The effect of omega-3 fatty acid supplementation on seizure frequency in individuals with epilepsy: a systematic review and meta-analysis. Nutr Neurosci.

[99] Reddy DS (2022). Therapeutic and clinical foundations of cannabidiol therapy for difficult-to-treat seizures in children and adults with refractory epilepsies. Exp Neurol.

[100] Zhu Z, Mittal R, Walser SA, Lehman E, Kumar A, Paudel S (2022). Complementary and Alternative Medicine (CAM) use in Children with Epilepsy. J Child Neurol.

[101] Kanhere SV, Bagadia DR, Phadke VD, Mukherjee PS (2018). Yoga in Children with Epilepsy: A Randomized Controlled Trial. J Pediatr Neurosci.

[102] Walker JE, Kozlowski GP. Neurofeedback treatment of epilepsy. Child Adolesc Psychiatr Clin N Am. 2005;14:163–76, viii. 10.1016/j.chc.2004.07.009 15564057

[103] Nigro SE (2019). The Efficacy of Neurofeedback for Pediatric Epilepsy. Appl Psychophysiol Biofeedback.

[104] Yucha, C., & Gilbert, C. Evidence-based practice in biofeed- back and neurotherapy. 2004.

[105] Höfer J, Hoffmann F, Bachmann C (2017). Use of complementary and alternative medicine in children and adolescents with autism spectrum disorder: A systematic review. Autism.

[106] Kamp-Becker I, Poustka L, Bachmann C, Ehrlich S, Hoffmann F, Kanske P (2017). Study protocol of the ASD-Net, the German research consortium for the study of Autism Spectrum Disorder across the lifespan: from a better etiological understanding, through valid diagnosis, to more effective health care. BMC Psychiatry.

[107] Lord C, Brugha TS, Charman T, Cusack J, Dumas G, Frazier T (2020). Autism spectrum disorder. Nat Rev Dis Primers.

[108] National Institute of Mental Health. A Parent’s Guide to Autism Spectrum Disorders. 2013. Available from: http://www.nimh.nih.gov/health/publications/a-parentsguide- to-autism-spectrum-disorder/index.shtml Accessed February 2, 2011.)

[109] Jobski K, Höfer J, Hoffmann F, Bachmann C (2017). Use of psychotropic drugs in patients with autism spectrum disorders: a systematic review. Acta Psychiatr Scand.

[110] Levy SE, Hyman SL (2008). Complementary and Alternative Medicine Treatments for Children with Autism Spectrum Disorders. Child Adolesc Psychiatr Clin N Am.

[111] Grimaldi R, Gibson GR, Vulevic J, Giallourou N, Castro-Mejía JL, Hansen LH (2018). A prebiotic intervention study in children with autism spectrum disorders (ASDs). Microbiome.

[112] Li B, Xu Y, Pang D, Zhao Q, Zhang L, Li M (2022). Interrelation between homocysteine metabolism and the development of autism spectrum disorder in children. Front Mol Neurosci.

[113] Campion D, Ponzo P, Alessandria C, Saracco GM, Balzola F (2018). The role of microbiota in autism spectrum disorders. Minerva Gastroenterol Dietol.

[114] Sivamaruthi BS, Suganthy N, Kesika P, Chaiyasut C (2020). The Role of Microbiome, Dietary Supplements, and Probiotics in Autism Spectrum Disorder. Int J Environ Res Public Health.

[115] Do, R.; Roberts, E.; Sichel, L.S.; Sichel, J. Improvements in gastrointestinal symptoms among children with autism spectrum disorder receiving the Delpro® probiotic and immunomodulator formulation. J Probiotics Health. 2013;

[116] Grossi E, Melli S, Dunca D, Terruzzi V. Unexpected improvement in core autism spectrum disorder symptoms after long-term treatment with probiotics. SAGE Open Med Case Rep. 2016;4:2050313X16666231. 10.1177/2050313X16666231 PMC5006292 27621806

[117] Cieślińska A, Sienkiewicz-Szłapka E, Wasilewska J, Fiedorowicz E, Chwała B, Moszyńska-Dumara M (2015). Influence of candidate polymorphisms on the dipeptidyl peptidase IV and μ-opioid receptor genes expression in aspect of the β-casomorphin-7 modulation functions in autism. Peptides.

[118] Shattock P, Whiteley P (2002). Biochemical aspects in autism spectrum disorders: updating the opioid-excess theory and presenting new opportunities for biomedical intervention. Expert Opin Ther Targets.

[119] Knivsberg AM, Reichelt KL, Høien T, Nødland M (2002). A randomised, controlled study of dietary intervention in autistic syndromes. Nutr Neurosci.

[120] González-Domenech PJ, Diaz-Atienza F, Gutiérrez-Rojas L, Fernández-Soto ML, González-Domenech CM (2022). A Narrative Review about Autism Spectrum Disorders and Exclusion of Gluten and Casein from the Diet. Nutrients.

[121] James S, Montgomery P, Williams K. Omega-3 fatty acids supplementation for autism spectrum disorders (ASD). Cochrane Database Syst Rev. 2011;CD007992. 10.1002/14651858.CD007992.pub2 PMC13606348 22071839

[122] Borgi M, Loliva D, Cerino S, Chiarotti F, Venerosi A, Bramini M (2016). Effectiveness of a Standardized Equine-Assisted Therapy Program for Children with Autism Spectrum Disorder. J Autism Dev Disord.

[123] Chen S, Zhang Y, Zhao M, Du X, Wang Y, Liu X (2022). Effects of Therapeutic Horseback-Riding Program on Social and Communication Skills in Children with Autism Spectrum Disorder: A Systematic Review and Meta-Analysis. Int J Environ Res Public Health.

[124] Pritchard S (2015). Tui Na: A Manual of Chinese Massage Therapy.

[125] Morhenn V, Beavin LE, Zak PJ (2012). Massage increases oxytocin and reduces adrenocorticotropin hormone in humans. Altern Ther Health Med.

[126] Li Q, Becker B, Wernicke J, Chen Y, Zhang Y, Li R (2019). Foot massage evokes oxytocin release and activation of orbitofrontal cortex and superior temporal sulcus. Psychoneuroendocrinology.

[127] Ruan H, Eungpinichpong W, Wu H, Shen M, Zhang A (2022). Medicine Insufficient Evidence for the Efficacy of Massage as Intervention for Autism Spectrum Disorder: A Systematic Review. Evid Based Complement Alternat Med.

[128] Wong VCN (2009). Use of complementary and alternative medicine (CAM) in autism spectrum disorder (ASD): comparison of Chinese and western culture (Part A). J Autism Dev Disord.

[129] Wang L, Peng J-L, Qiao F-Q, Cheng W-M, Lin G-W, Zhang Y (2021). Clinical Randomized Controlled Study of Acupuncture Treatment on Children with Autism Spectrum Disorder (ASD): A Systematic Review and Meta-Analysis. Evid Based Complement Alternat Med.

[130] Jensen M-L, Vamosi M. The association between nonpharmacological interventions and quality of life in children with attention deficit hyperactivity disorder: A systematic review. J Child Adolesc Psychiatr Nurs. 2022; 10.1111/jcap.12402 36380398

[131] Spencer T, Biederman J, Wilens T (2000). Pharmacotherapy of attention deficit hyperactivity disorder. Child Adolesc Psychiatr Clin N Am.

[132] Wu J, Li P, Luo H, Lu Y (2022). Complementary and Alternative Medicine Use by ADHD Patients: A Systematic Review. J Atten Disord.

[133] Sinha D, Efron D (2005). Complementary and alternative medicine use in children with attention deficit hyperactivity disorder. J Paediatr Child Health.

[134] United States Pharmacopeial Convention (2000). Drug Information for the Health Care Professional.

[135] Johnstone JM, Hatsu I, Tost G, Srikanth P, Eiterman LP, Bruton AM (2022). Micronutrients for Attention-Deficit/Hyperactivity Disorder in Youths: A Placebo-Controlled Randomized Clinical Trial. J Am Acad Child Adolesc Psychiatry.

[136] Ni X, Zhang-James Y, Han X, Lei S, Sun J, Zhou R (2014). Traditional Chinese medicine in the treatment of ADHD: a review. Child Adolesc Psychiatr Clin N Am.

[137] Ni X, Zhang JY, Han X (2015). Yin D [A Meta-analysis on Acupuncture Treatment of Attention Deficit/Hyperactivity Disorder]. Zhen Ci Yan Jiu.

[138] Xing L, Ren Z, Yue X, Chen H, Xia C, Liu F (2021). Acupuncture treatment on attention deficit hyperactivity disorder: A protocol for systematic review and meta-analysis. Medicine.

[139] Gaertner K, Teut M, Walach H. Is homeopathy effective for attention deficit and hyperactivity disorder? A meta-analysis. Pediatr Res. 2022; 10.1038/s41390-022-02127-3 35701608

[140] Sampedro Baena L, Fuente GAC-D la, Martos-Cabrera MB, Gómez-Urquiza JL, Albendín-García L, Romero-Bejar JL, et al. Effects of Neurofeedback in Children with Attention-Deficit/Hyperactivity Disorder: A Systematic Review. J Clin Med. 2021;10:3797. 10.3390/jcm10173797 PMC8432262 34501246

[141] Stern P, Shalev L (2013). The role of sustained attention and display medium in reading comprehension among adolescents with ADHD and without it. Res Dev Disabil.

[142] Chen I-C, Chan H-Y, Lin K-C, Huang Y-T, Tsai P-L, Huang Y-M (2022). Listening to White Noise Improved Verbal Working Memory in Children with Attention-Deficit/Hyperactivity Disorder: A Pilot Study. IJERPH.

[143] Zhu C (2022). Effects of Musicotherapy Combined with Cognitive Behavioral Intervention on the Cognitive Ability of Children with Attention Deficit Hyperactivity Disorder. Psychiatr Danub.

[144] Frey J, Malaty IA (2022). Tourette Syndrome Treatment Updates: a Review and Discussion of the Current and Upcoming Literature. Curr Neurol Neurosci Rep.

[145] Seideman MF, Seideman TA (2020). A Review of the Current Treatment of Tourette Syndrome. J Pediatr Pharmacol Ther.

[146] Patel H, Nguyen K, Lehman E, Mainali G, Duda L, Byler D (2020). Use of Complementary and Alternative Medicine in Children With Tourette Syndrome. J Child Neurol.

[147] Pringsheim T, Okun MS, Müller-Vahl K, Martino D, Jankovic J, Cavanna AE (2019). Practice guideline recommendations summary: Treatment of tics in people with Tourette syndrome and chronic tic disorders. Neurology.

[148] Lu C, Wu L-Q, Hao H, Kimberly Leow X, Xu F-W, Li P-P (2021). Clinical efficacy and safety of acupuncture treatment of TIC disorder in children: A systematic review and meta-analysis of 22 randomized controlled trials. Complement Ther Med.

[149] Qi H, Liu R, Zheng W, Zhang L, Ungvari GS, Ng CH (2020). Efficacy and safety of traditional Chinese medicine for Tourette’s syndrome: A meta-analysis of randomized controlled trials. Asian J Psychiatr.

[150] Ding L, Yang Z, Liu G, Ran N, Yi M, Li H (2020). Safety and efficacy of taurine as an add-on treatment for tics in youngsters. Eur J Neurol.

[151] Bond M, Moll N, Rosello A, Bond R, Schnell J, Burger B (2022). Vitamin D levels in children and adolescents with chronic tic disorders: a multicentre study. Eur Child Adolesc Psychiatry.

[152] Seeff LB, Lindsay KL, Bacon BR, Kresina TF, Hoofnagle JH (2001). Complementary and alternative medicine in chronic liver disease. Hepatology.

[153] Verma S, Thuluvath PJ (2007). Complementary and alternative medicine in hepatology: review of the evidence of efficacy. Clin Gastroenterol Hepatol.

[154] Gelow K, Chalasani S, Green K, Lammert C (2022). Utilization and Impact of Complementary and Alternative Medicines in Symptomatic Autoimmune Hepatitis Patients. Dig Dis Sci.

[155] Guan Y-S, He Q (2013). A current update on the rule of alternative and complementary medicine in the treatment of liver diseases. Evid Based Complement Alternat Med.

[156] White CP, Hirsch G, Patel S, Adams F, Peltekian KM (2007). Complementary and alternative medicine use by patients chronically infected with hepatitis C virus. Can J Gastroenterol.

[157] Richmond JA, Bailey DE, Patel K, Jezsik JA, Muir A, Lin J-R (2010). The use of complementary and alternative medicine by patients with chronic hepatitis C. Complement Ther Clin Pract.

[158] Li QS, Cao SH, Xie GM, Gan YH, Ma HJ, Lu JZ, et al. Combined traditional Chinese medicine and Western medicine. Relieving effects of Chinese herbs, ear-acupuncture and epidural morphine on postoperative pain in liver cancer. Chin Med J (Engl). 1994;107:289–94. 8088198

[159] Batey RG, Salmond SJ, Bensoussan A (2005). Complementary and alternative medicine in the treatment of chronic liver disease. Curr Gastroenterol Rep.

[160] Pietrangelo A, Borella F, Casalgrandi G, Montosi G, Ceccarelli D, Gallesi D (1995). Antioxidant activity of silybin in vivo during long-term iron overload in rats. Gastroenterology.

[161] Dehmlow C, Erhard J, de Groot H (1996). Inhibition of Kupffer cell functions as an explanation for the hepatoprotective properties of silibinin. Hepatology.

[162] Campos R, Garrido A, Guerra R, Valenzuela A (1989). Silybin dihemisuccinate protects against glutathione depletion and lipid peroxidation induced by acetaminophen on rat liver. Planta Med.

[163] de Avelar CR, Pereira EM, de Farias Costa PR, de Jesus RP, de Oliveira LPM (2017). Effect of silymarin on biochemical indicators in patients with liver disease: Systematic review with meta-analysis. World J Gastroenterol.

[164] Boigk G, Stroedter L, Herbst H, Waldschmidt J, Riecken EO, Schuppan D (1997). Silymarin retards collagen accumulation in early and advanced biliary fibrosis secondary to complete bile duct obliteration in rats. Hepatology.

[165] Ferenci P, Dragosics B, Dittrich H, Frank H, Benda L, Lochs H (1989). Randomized controlled trial of silymarin treatment in patients with cirrhosis of the liver. J Hepatol.

[166] Salmi HA, Sarna S. Effect of silymarin on chemical, functional, and morphological alterations of the liver. A double-blind controlled study. Scand J Gastroenterol. 1982;17:517–21. 10.3109/00365528209182242 6753109

[167] Berkson BM. A conservative triple antioxidant approach to the treatment of hepatitis C. Combination of alpha lipoic acid (thioctic acid), silymarin, and selenium: three case histories. Med Klin (Munich). 1999;94 Suppl 3:84–9. 10.1007/BF03042201 10554539

[168] Tanamly MD, Tadros F, Labeeb S, Makld H, Shehata M, Mikhail N (2004). Randomised double-blinded trial evaluating silymarin for chronic hepatitis C in an Egyptian village: study description and 12-month results. Dig Liver Dis.

[169] Bode JC, Schmidt U (1977). Dürr HK [Silymarin for the treatment of acute viral hepatitis? Report of a controlled trial (author’s transl)]. Med Klin.

[170] Angulo P, Patel T, Jorgensen RA, Therneau TM, Lindor KD (2000). Silymarin in the treatment of patients with primary biliary cirrhosis with a suboptimal response to ursodeoxycholic acid. Hepatology.

[171] Hruby K, Csomos G, Fuhrmann M, Thaler H (1983). Chemotherapy of Amanita phalloides poisoning with intravenous silibinin. Hum Toxicol.

[172] Patrick L (1999). Hepatitis C: epidemiology and review of complementary/alternative medicine treatments. Altern Med Rev.

[173] van Rossum TG, Vulto AG, de Man RA, Brouwer JT, Schalm SW (1998). Review article: glycyrrhizin as a potential treatment for chronic hepatitis C. Aliment Pharmacol Ther.

[174] Shaikh ZA, Vu TT, Zaman K (1999). Oxidative stress as a mechanism of chronic cadmium-induced hepatotoxicity and renal toxicity and protection by antioxidants. Toxicol Appl Pharmacol.

[175] Wang JY, Guo JS, Li H, Liu SL, Zern MA (1998). Inhibitory effect of glycyrrhizin on NF-kappaB binding activity in CCl4- plus ethanol-induced liver cirrhosis in rats. Liver.

[176] Yoshikawa M, Matsui Y, Kawamoto H, Umemoto N, Oku K, Koizumi M (1997). Effects of glycyrrhizin on immune-mediated cytotoxicity. J Gastroenterol Hepatol.

[177] Stati G, Rossi F, Sancilio S, Basile M, Di Pietro R. Curcuma longa Hepatotoxicity: A Baseless Accusation. Cases Assessed for Causality Using RUCAM Method. Front Pharmacol. 2021;12:780330. 10.3389/fphar.2021.780330 PMC8586077 34776989

[178] Buonomo AR, Scotto R, Nappa S, Arcopinto M, Salzano A, Marra AM (2019). The role of curcumin in liver diseases. Arch Med Sci.

[179] Zeng Y, Luo Y, Wang L, Zhang K, Peng J, Fan G (2023). Therapeutic Effect of Curcumin on Metabolic Diseases: Evidence from Clinical Studies. Int J Mol Sci.

[180] Osawa T, Sugiyama Y, Inayoshi M, Kawakishi S (1995). Antioxidative activity of tetrahydrocurcuminoids. Biosci Biotechnol Biochem.

[181] Farzaei MH, Zobeiri M, Parvizi F, El-Senduny FF, Marmouzi I, Coy-Barrera E (2018). Curcumin in Liver Diseases: A Systematic Review of the Cellular Mechanisms of Oxidative Stress and Clinical Perspective. Nutrients.

[182] Jalali M, Mahmoodi M, Mosallanezhad Z, Jalali R, Imanieh MH, Moosavian SP (2020). The effects of curcumin supplementation on liver function, metabolic profile and body composition in patients with non-alcoholic fatty liver disease: A systematic review and meta-analysis of randomized controlled trials. Complement Ther Med.

[183] Williamson EM (2001). Synergy and other interactions in phytomedicines. Phytomedicine.

[184] Chang IM (1998). Liver-protective activities of aucubin derived from traditional oriental medicine. Res Commun Mol Pathol Pharmacol.

[185] Jeong H-J, Koo H-N, Na H-J, Kim M-S, Hong S-H, Eom J-W (2002). Inhibition of TNF-alpha and IL-6 production by Aucubin through blockade of NF-kappaB activation RBL-2H3 mast cells. Cytokine.

[186] Wang L, Wang B-E, Wang J, Xiao P-G, Tan X-H (2008). Herbal compound 861 regulates mRNA expression of collagen synthesis- and degradation-related genes in human hepatic stellate cells. World J Gastroenterol.

[187] You H, Wang B (2000). Wang T [Proliferation and apoptosis of hepatic stellate cells and effects of compound 861 on liver fibrosis]. Zhonghua Gan Zang Bing Za Zhi.

[188] Oka H, Yamamoto S, Kuroki T, Harihara S, Marumo T, Kim SR (1995). Prospective study of chemoprevention of hepatocellular carcinoma with Sho-saiko-to (TJ-9). Cancer.

[189] Yamashiki M, Nishimura A, Nomoto M, Suzuki H, Kosaka Y (1996). Herbal medicine “Sho-saiko-to” induces tumour necrosis factor-alpha and granulocyte colony-stimulating factor in vitro in peripheral blood mononuclear cells of patients with hepatocellular carcinoma. J Gastroenterol Hepatol.

[190] Schuppan D, Jia JD, Brinkhaus B, Hahn EG (1999). Herbal products for liver diseases: a therapeutic challenge for the new millennium. Hepatology.

[191] Chan E, Rappaport LA, Kemper KJ (2003). Complementary and alternative therapies in childhood attention and hyperactivity problems. J Dev Behav Pediatr.

[192] Rietveld A, Wiseman S (2003). Antioxidant effects of tea: evidence from human clinical trials. J Nutr.

[193] Grimble RF, Grimble GK (1998). Immunonutrition: role of sulfur amino acids, related amino acids, and polyamines. Nutrition.

[194] Rambaldi A, Gluud C. S-adenosyl-L-methionine for alcoholic liver diseases. Cochrane Database Syst Rev. 2006;CD002235. 10.1002/14651858.CD002235.pub2 16625556

[195] Harrison SA, Torgerson S, Hayashi P, Ward J, Schenker S (2003). Vitamin E and vitamin C treatment improves fibrosis in patients with nonalcoholic steatohepatitis. Am J Gastroenterol.

[196] Ott M, Thyagarajan SP, Gupta S (1997). Phyllanthus amarus suppresses hepatitis B virus by interrupting interactions between HBV enhancer I and cellular transcription factors. Eur J Clin Invest.

[197] Jeong T-Y, Park B-K, Cho J-H, Kim Y-I, Ahn Y-C, Son C-G (2012). A prospective study on the safety of herbal medicines, used alone or with conventional medicines. J Ethnopharmacol.

[198] Center SA (2004). Metabolic, antioxidant, nutraceutical, probiotic, and herbal therapies relating to the management of hepatobiliary disorders. Vet Clin North Am Small Anim Pract.

[199] Gunawan B, Kaplowitz N (2004). Clinical perspectives on xenobiotic-induced hepatotoxicity. Drug Metab Rev.

